# Wafer-scale fabrication and growth dynamics of suspended graphene nanoribbon arrays

**DOI:** 10.1038/ncomms11797

**Published:** 2016-06-02

**Authors:** Hiroo Suzuki, Toshiro Kaneko, Yasushi Shibuta, Munekazu Ohno, Yuki Maekawa, Toshiaki Kato

**Affiliations:** 1 Department of Electronic Engineering, Tohoku University, Aoba 6-6-05, Aramaki, Aoba-ku, Sendai 980-8579, Japan; 2 Department of Materials Engineering, The University of Tokyo, 7-3-1, Hongo, Bunkyo-ku, Tokyo 113-8656, Japan; 3 Division of Materials Science and Engineering, Faculty of Engineering, Hokkaido University, Kita 13 Nishi 8, Kita-ku, Sapporo, Hokkaido 060-8628, Japan

## Abstract

Adding a mechanical degree of freedom to the electrical and optical properties of atomically thin materials can provide an excellent platform to investigate various optoelectrical physics and devices with mechanical motion interaction. The large scale fabrication of such atomically thin materials with suspended structures remains a challenge. Here we demonstrate the wafer-scale bottom–up synthesis of suspended graphene nanoribbon arrays (over 1,000,000 graphene nanoribbons in 2 × 2 cm^2^ substrate) with a very high yield (over 98%). Polarized Raman measurements reveal graphene nanoribbons in the array can have relatively uniform-edge structures with near zigzag orientation dominant. A promising growth model of suspended graphene nanoribbons is also established through a comprehensive study that combined experiments, molecular dynamics simulations and theoretical calculations with a phase-diagram analysis. We believe that our results can contribute to pushing the study of graphene nanoribbons into a new stage related to the optoelectrical physics and industrial applications.

## Abstract (web summary)

Shaping atomically thin materials in suspended structures may provide a viable platform for nanoscale mechanical oscillators. Here, the authors demonstrate wafer-scale, high-yield synthesis of suspended graphene nanoribbon arrays using a bottom-up approach and shed light into their growth dynamics.

## Graphical abstract

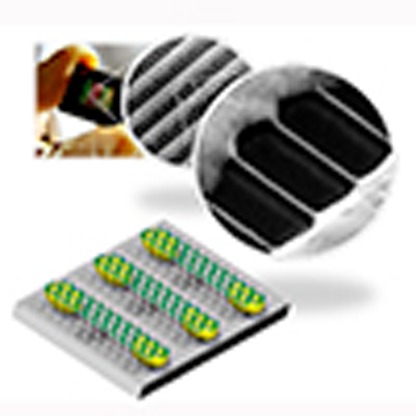

Atomically thin materials such as graphene^1^,^2^ and other two-dimensional (2D) materials^3^,^4^ attract intense attention because of their prominent electrical and optical properties^5^,^6^,^7^. Because of their extraordinarily small thickness, large surface area, low mass density and high Young's modulus, atomically thin materials also have excellent mechanical properties and can be used as good detectors of mass, force and charge^8^,^9^. Combining these superior optoelectrical features with their excellent mechanical properties can open up the possibility of utilizing atomically thin materials in wide variety of scientific fields and applications. To use the mechanical degree of freedom, a freely suspended structure allowing mechanical vibration is necessary. Thus, the fabrication and integration of atomically thin materials with suspended structures is one of the most important subjects from both fundamental and industrial points of view. Up to now, various suspended devices with nanoscale materials have been demonstrated, including single-walled carbon nanotubes^10^,^11^, graphene^12^,^13^, nanowire^14^ and other 2D materials.^15^,^16^, resulting in many promising features such as the quantum-coherent coupling of a mechanical oscillator with optical cavities^17^, current-induced black-body radiation^18^, ballistic interference^19^ and a nanoscale mechanical resonator^20^. Graphene nanoribbon (GNR), which consists of strips of graphene, is another candidate for such atomically thin materials. It has a high carrier mobility, clear band gap, and spin-polarized edge state, which do not appear in other materials^21^,^22^,^23^,^24^. This indicates that suspended GNR can provide a promising platform for a wide variety of scientific fields and applications.

The preparation of GNRs is now possible using both top–down and bottom–up approaches. GNRs with high-width controllability can be fabricated by etching 2D graphene^21^,^25^,^26^. The unzipping of carbon nanotube can prepare high-quality GNR^27^,^28^,^29^. The decomposition of SiC can realize the integrated synthesis of GNRs^30^,^31^. The edge structures can be controlled through the organic synthesis of GNRs^32^,^33^. However, these GNR fabrication methods are limited to on-substrate structures. There are few methods for fabricating suspended GNRs, which are the combination of 2D graphene (carbon nanotube) etching (unzipping) and partial etching of the SiO_2_ substrate under GNRs to bridge the GNRs between electrodes^34^. This top–down approach is limited to a small sample area, and causes significant damage, which results in a low device performance. Recently, we developed a novel bottom–up synthesis method for suspended GNRs that combines a Ni nanobar catalyst and advanced plasma processing^35^. Site and alignment-controlled GNR growth with a suspended form can be realized. Although this technique has huge potential for the integration of suspended GNR devices, the GNR growth yield is very low, which strongly restricts its utilization in various studies. The unknown growth mechanism also makes it difficult to control the structures of the suspended GNRs, which is the critical issue to obtain better device performance.

In this study, we solve these critical issues and realize a wafer-scale (2 × 2 cm^2^) high-yield synthesis (over 98%) of suspended GNR arrays (∼1,000,000 GNRs). Polarized Raman mapping measurements reveal the suspended GNR array includes uniform D-band to G-band intensity ratio with parallel polarization (*I*_D//_ over *I*_G//_=0.95±0.25), which should denote the uniform-edge structure with near zigzag rich orientation. Local-gate operation with this suspended GNR is also demonstrated, showing that various electrically addressable logic circuits can be fabricated on a large scale using this suspended GNR. A short (below 100 nm) GNR with a suspended structure shows very high electrical conductivity (approximately 10^6^ Sm^−1^). The growth kinetics of the suspended GNR is also systematically investigated through a comparison of thermal chemical vapour deposition (CVD) and plasma CVD results. Theoretical calculations and molecular dynamics simulations are also used to identify the detailed nucleation dynamics of the suspended GNR. Locally gate-tunable suspended GNR arrays with high electrical conductivity can open up a novel stage for both fundamental studies and practical applications of GNR in various optoelectrical, chemical and biological application fields in combination with the mechanical degree of freedom.

## Results

### Wafer-scale high-yield synthesis of suspended GNRs

**Figure 1:**
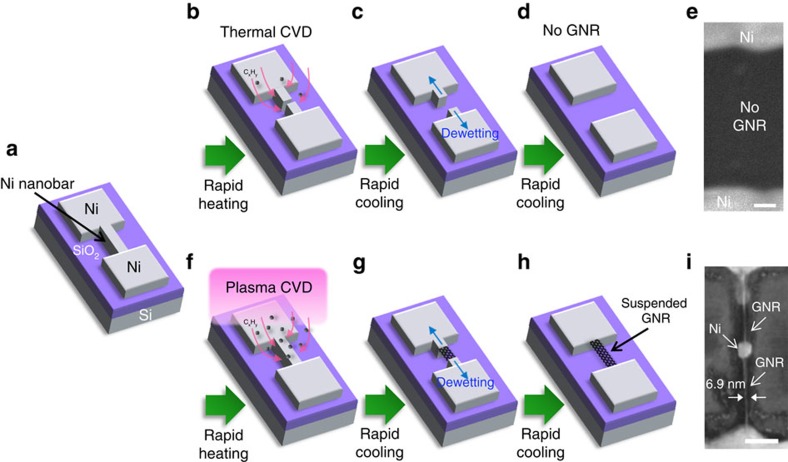
Comparison of growth method between thermal CVD and Plasma CVD. (**a**–**d**,**f**–**h**) Schematic illustration of growth method for suspended GNR by (**a**–**d**) rapid heating thermal CVD and (**a**,**f**–**h**) rapid heating plasma CVD (RH-PCVD). Arrows in **b**,**f** and **c**,**g** shows the direction of hydrocarbon (C_*x*_H_*y*_) supply and dewetting of Ni nanobar, respectively. (**e**,**i**) Typical SEM images of the (**e**) broken Ni nanobar and (**i**) suspended GNR with sub 10 nm width. Scale bar, 100 nm (**e**,**i**).

The synthesis of GNRs was carried out using the previously developed rapid-heating plasma CVD (RH-PCVD)^35^. The typical fabrication process is shown in Fig. 1. A Ni nanobar (width ranging from 20 to 100 nm) was fabricated with large Ni electrodes using conventional electron beam lithography and a lift-off process followed by Ni deposition (thickness: ∼80 nm) (Fig. 1a; see Methods section for more detailed conditions). The systematic comparison of plasma and thermal CVD was possible by simply turning on and off the radio frequency (13.56 MHz) power supplied to the coils placed outside a quartz tube. In the case of thermal CVD, the Ni nanobar was broken, and GNRs could not be grown (Fig. 1b–e), whereas suspended GNRs could be formed between the Ni electrodes using plasma CVD (Fig. 1f–i; the detailed growth dynamics will be discussed later).

**Figure 2:**
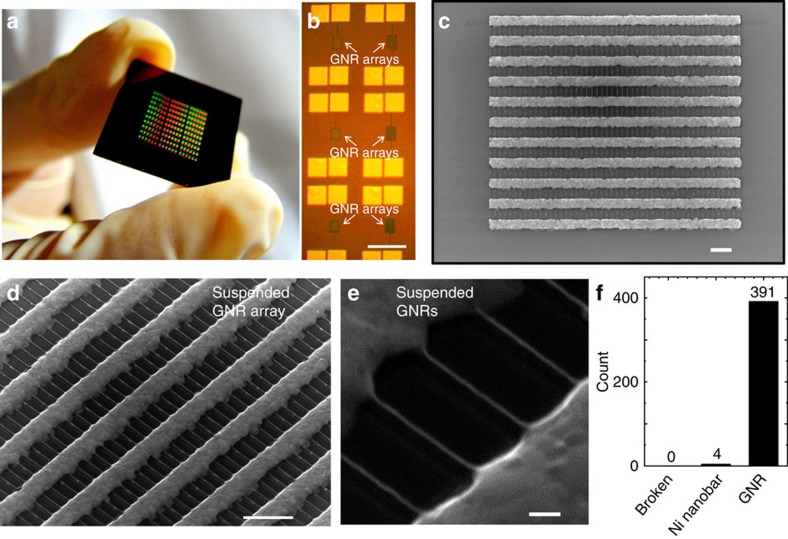
Wafer-scale synthesis of suspended GNR arrays. (**a**–**c**) (**a**) low- and (**b**) high-magnification optical microscope image and (**c**) SEM image of wafer-scale synthesis of suspended GNR arrays. Scale bar, 200 μm (**b**); 1 μm (**c**). (**d**,**e**) Typical (**d**) low- and (**e**) high-magnification SEM images of suspended GNRs grown under the high growth yield. Scale bar, 1 μm (**d**); 100 nm (**e**). (**f**) Histogram of product concentrations in suspended GNR array (**d**). We counted the broken structure of Ni without GNR, Ni nanobar without GNR and GNRs regardless of the amount of Ni nanostructures remained as Broken, Ni nanobar and GNR, respectively. The count in **f** represents the number of each structure. The total amount of counted structure is 395.

To improve the growth yield of suspended GNRs, we optimized the various growth conditions such as the growth temperature, temperature increasing rate, total process time, cooling rate, gas pressure, gas mixture ratio, plasma generation power and plasma irradiation time. The growth yield of GNRs was estimated using the GNR conversion rate from Ni nanobar in dense array structures. Each Ni nanobar space was set at 250 nm. Supplementary Fig. 1a shows the growth yield of suspended GNRs as a function of the plasma irradiation time (*t*_p_), which is one of the most sensitive growth parameters. In region A, where the growth yield is low, a broken Ni nanobar was often observed (Supplementary Fig. 1b). In contrast, in region C, the amount of Ni residue was high (Supplementary Fig. 1d). The high-yield synthesis of suspended GNRs could only be realized between these conditions in region B (Supplementary Fig. 1a,c; Supplementary Note 1). This narrow growth window and high sensitivity to *t*_p_ could be well explained by the growth model established in this study (see Discussion section). Under suitable conditions, we succeeded in the fabrication of GNR arrays with a high growth yield of >98% in a wafer-scale substrate (totally over 1,000,000 suspended GNRs on 2 × 2 cm^2^ substrate; Fig. 2a–f). This is the first time that the formation of suspended GNR arrays has been realized on a wafer-scale (Supplementary Fig. 2; Supplementary Note 2). The sub-10-nm order (minimum: ∼6.9 nm) of narrow GNRs can be also grown by our method (Supplementary Fig. 3; Supplementary Note 3), which is almost same order of GNRs produced by the other method both for top–down and bottom–up approaches^21^,^25^,^26^,^27^,^28^,^29^,^30^,^31^,^32^,^33^,^34^.

### Edge-structure analysis of suspended GNRs arrays

**Figure 3:**
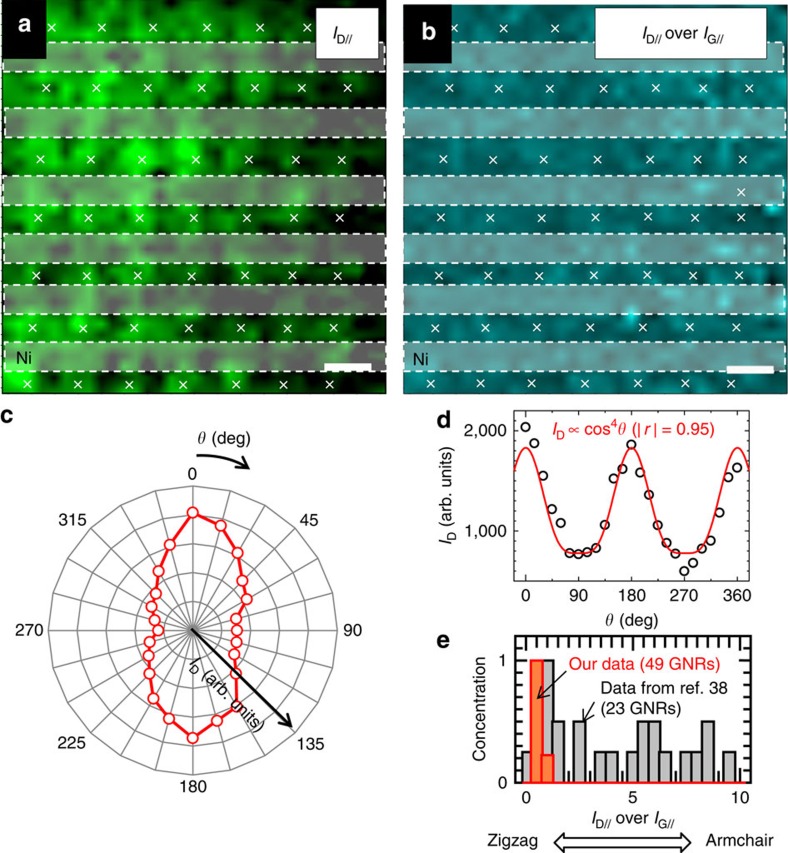
Polarized Raman features of suspended GNR array. (**a**–**e**) Integrated intensity mapping of (**a**) *I*_D//_ and (**b**) *I*_D//_ over *I*_G//_ of suspended GNR array. The sign of **×** in **a** and **b** shows the position of each GNR. Scale bar, 1 μm (**a**,**b**). (**c**) Polar plot of *I*_D_. (**d**) *I*_D_ as a function of *θ*. (**e**) Histogram of *I*_D//_ over *I*_G//_ for our GNRs and unzipped GNR (traced from ref. 38). The smaller (larger) value of *I*_D//_ over *I*_G//_ corresponds to zigzag (armchair) dominant edge orientation as descrived with arrow in **e**.

The edge-structure control of GNRs is regarded as one of the most important subject as can be seen in several recent progresses with organic synthesis^36^ and epitaxial synthesis of GNRs on a Ge substrate^37^. Then, the edge structure of suspended GNR arrays was carefully investigated with polarized Raman mapping measurements, where the orientation of the polarizer for inspecting the scattered light was parallel to the polarization of the incident light (VV configuration; see Methods section for detailed measurement set-up). Bright and dark patterns can be appeared in D-band intensity mapping with parallel polarization (*I*_D//_), which well matches with the location of GNRs confirmed by SEM, denoting the polarized Raman signal can be obtained from each individual GNR (Fig. 3a,b). Plot of *I*_D//_ against to the polarized angle (*θ*) shows periodic oscillation, which can be fitted with a function of cos^4^*θ* (Fig. 3c,d). This is consistent with previous reports^38^. Polarized Raman features can be also obtained from G-band (Supplementary Fig. 4; Supplementary Note 4). To identify the distribution of edge orientation of GNRs in the array, the distribution of D/G ratio with parallel polarization (*I*_D//_ over *I*_G//_) was measured for 49 GNRs in the same array. The distribution of *I*_D//_ over *I*_G//_ is very narrow compared with previously reported value by other group with unzipped carbon nanotubes^38^, denoting relatively uniform-edge structures should be formed in our GNR array (Fig. 3b,e). The averaged *I*_D//_ over *I*_G//_ is about 0.95±0.25 showing the edge can be near zigzag structures (Fig. 3e). The relatively small D-band intensity ratio of parallel to perpendicular polarization (*I*_D//_ over *I*_D⊥_) as can be seen in Fig. 3c may be due to the microscopic misalignment or amorphous carbon residue (Supplementary Fig. 5; Supplementary Note 4).

Although the concrete mechanism of preferential synthesis of near-zigzag edge orientation is still not clear, the possible explanation can be given by considering the energy balance between armchair and zigzag edges. It has been reported the there is a clear difference in the energy required to vaporize a C–C unit from the armchair edge (∼6.7 eV) and zigzag edge (∼11 eV)^39^. The preferential stability of zigzag edges has also been reported by other groups^40^,^41^. Since the growth reaction is happened during the high-temperature condition (∼900 °C) the bond structure of edge should be renormalized during or after the nucleation process, resulting in the stable structure dominant, which should be the zigzag or near zigzag edges. Although the thermal energy (900 °C≃0.1 eV) itself is not enough to cause the bond renormalization, the catalytic effect of Ni can lower the threshold energy for the bond renormalization. The suspended structure of our GNR sample can also enhance the renormalization of edge structures. When GNRs attach on the substrate, the required energy to cause bond renormalization should be higher than that of suspended one due to the surface interaction between substrate and carbon atoms at the GNR edges. Thus, the free-standing structure of GNR edge can be another important point to realize GNR synthesis with zigzag or near zigzag edge dominant orientation.

### Electrical properties of suspended GNR

**Figure 4:**
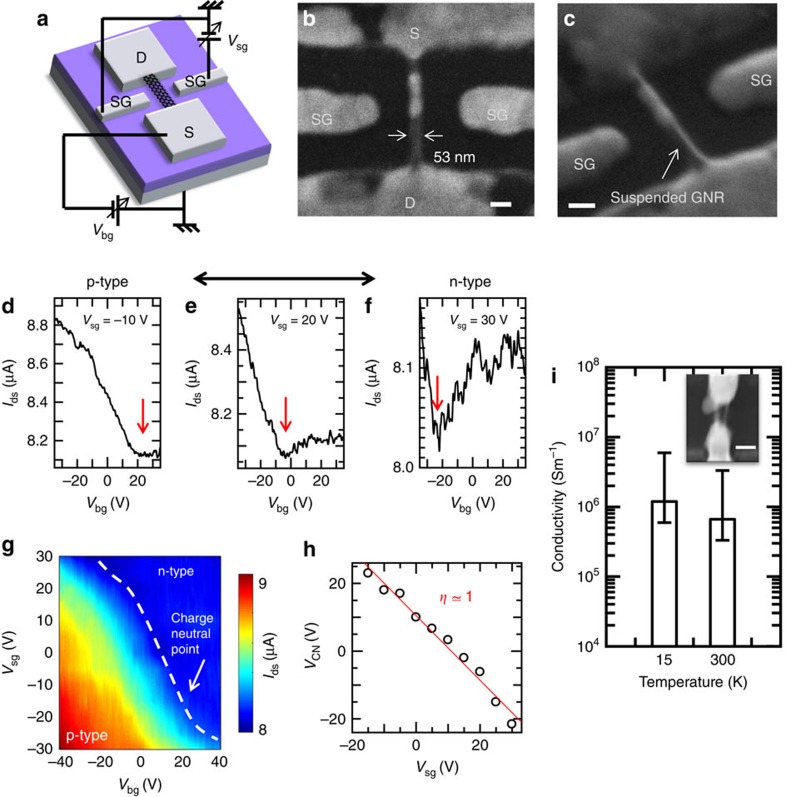
Side-gate operation of suspended GNRs device. (**a**–**c**) (**a**) Schematic illustration and (**b**,**c**) SEM images of side-gate suspended GNR transistor. Scale bar, 100 nm (**b**,**c**). (**d**–**f**) *I*_ds_–*V*_bg_ curves of suspended GNR transistor under different *V*_sg_ bias voltages ((**d**) *V*_sg_=−10 V, (**e**) 20 V, (**f**) 30 V). The red and black arrows in **d**–**f** show the position of charge neutral point and transition of carrier type between p and n, respectively. (**g**) Contour plot of *I*_ds_ as function of *V*_bg_ and *V*_sg_. (**h**) Plot of *V*_CN_ varying with *V*_sg_. Red line in **h** shows the linear fitting line. (**i**) Conductivity of short suspended GNR under 15 and 300 K. Inset in **i** denotes the SEM image of short suspended GNR; Scale bar, 100 nm. The error bars correspond to the range of conductivity difference calculated with different thickness of GNR from 1 to 10 nm, that is minimum and maximum value in each error bar corresponds to the calculated conductivity assuming GNR thickness as 10 and 1 nm, respectively.

This high-yield synthesis of suspended GNRs will make it possible to design various electrical device structures. Since an electrically addressable local-gate system is necessary to develop complementary metal-oxide semiconductor circuits, we attempted to fabricate a local-gate transistor with suspended GNR. Fig. 4a–c shows a schematic illustration (Fig. 4a) and scanning electron microscope (SEM) images (Fig. 4b,c) of a suspended GNR transistor with side-gate structures. Note that the side-gate electrodes were prepared with Ni nanobar before the plasma CVD, indicating one step fabrication of side-gate structures without any post processes is possible. In the case of a side-gate bias voltage (*V*_sg_)=−10 V, the drain–source current (*I*_ds_) could be tuned by the back-gate voltage (*V*_bg_), showing the well-known *I*_ds_–*V*_bg_ feature with a charge neutral point (*V*_CN_) at *V*_gs_=22 V (Fig. 4d). The charge neutral point could be shifted to −5 and −22 V by changing *V*_sg_ to 20 and 30 V, respectively (Fig. 4e,f). The contour plot of *I*_sd_ as functions of *V*_bg_ and *V*_sg_ also showed the clear dependence of the charge neutral point modulation by *V*_sg_ (Fig. 4g). The linear fitting performed for *V*_CN_ as a function of *V*_sg_ revealed a side-gate efficiency (*η*=*ΔV*_CN_/*ΔV*_sg_) of *η*∼1 (Fig. 4h). This indicated that the side-gate can locally control the conductivity of the suspended GNR. Similar features can be also obtained with the other GNR device with side-gate structures (Supplementary Fig. 6; Supplementary Note 5). To the best of our knowledge, this is the first demonstration of the local-gate operation of a suspended GNR transistor. A very high electrical conductivity could also be observed for the relatively short suspended GNR. The conductivity of the short-channel (∼77 nm) suspended GNR (width: ∼50 nm) was about 10^6^ Sm^−1^ at 15 K (Fig. 4i). This value was almost the same order or slightly higher than that of a GNR grown by unzipping carbon nanotube^29^, which is known to produce a very high-quality GNR. Because of the difficulty of thickness measurement of suspended GNR, the thickness of GNR was assumed as 5 nm for the conductivity calculation. The variation of conductivity calculated with different thickness from 1 to 10 nm was also shown as error bars in Fig. 4i. Note that the electrical performance in this study was measured using a two-point contact measurement because of the unique device structure, implying that the intrinsic conductivity of the suspended GNRs may be far higher than that obtained by this two-point measurement.

As we demonstrated, our fabrication method for suspended GNRs includes the following benefits: first, wafer-scale integration; second, relatively uniform-edge structure with near zigzag orientation; third, electrically addressable local-gate structures; and fourth, high electrical conductivity. These can contribute to pushing GNR studies from basic science to practical use, especially in the optoelectrical field for various sensors (gas, chemical and biological), highly sensitive photon detectors in the terahertz region, and also the spintronic device application using unique spin state in zigzag edge GNRs in combination with the mechanical motion of GNRs.

### Growth dynamics of suspended GNR

We demonstrated the successful fabrication of highly conductive suspended GNR arrays on a wafer-scale. Further adjustments in the GNR structures such as the width, length and layer number can open up the application field of this novel device. To realize the fine tuning of GNR structures, it is necessary to thoroughly understand the detailed growth mechanism of suspended GNRs.

In general, there are two different growth modes in graphene, depending on the catalyst. In case of a catalyst with low carbon solubility such as Cu, the supplied carbon migrates on the catalyst surface, and graphene grows along the surface of the catalyst from the nucleation center. This is called the surface diffusion model and is known as an isothermal reaction^42^,^43^. On the other hand, in case of a catalyst with high carbon solubility such as Ni, the supplied carbon penetrates into the catalyst. Thereafter, the graphene is grown during the cooling process by the precipitation of carbon, which is called the bulk diffusion model and is governed by a non-isothermal reaction^43^.

Because we used a nanoscale Ni catalyst (Ni nanobar) in this study, we basically used the non-isothermal model to describe the GNR growth in our method. Although the isothermal reaction can occur even with Ni under low-temperature (∼600 °C) growth^44^, the temperature range used in this study was much higher (∼900 °C). To explain the experimental result, that is, suspended GNRs being formed from Ni nanobar, the following two required conditions should be considered. First, the Ni nanobar should maintain its fine structure even under a high-temperature condition (∼900 °C), at least until just before the cooling process. Second, the Ni nanobar should be spatially dissipated, followed by the GNR growth during the cooling process.

To establish an accurate growth model satisfying the above two requirements, we carried out experiments by focusing on the following two essential questions. The one is for the reason why a Ni nanobar can maintain its fine structure even under high-temperature (∼900 °C) conditions. The other is about the formation mechanism of the suspended GNR structures during the cooling process.

**Figure 5:**
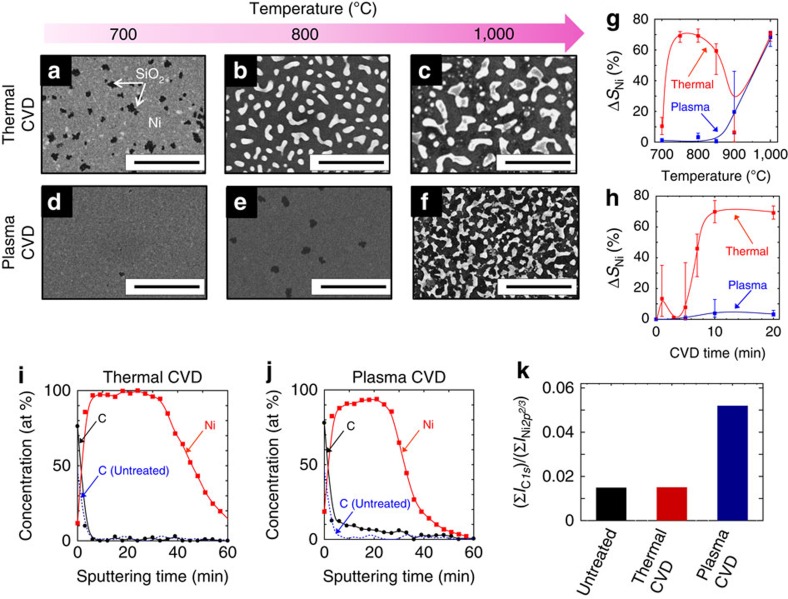
Comparison of Ni-film stability between thermal CVD and plasma CVD. (**a**–**f**) SEM images of Ni surface after (**a**–**c**) thermal CVD and (**d**–**f**) plasma CVD under various temperature conditions ((**a**,**d**) 700 °C, (**b**,**e**) 800 °C, (**c**,**f**) 1,000 °C). Scale bar, 5 μm (**a**–**f**). (**g**,**h**) Plot of Δ*S*_Ni_ as a function of (**g**) temperature and (**h**) CVD time. The red and blue colours in **g** and **h** show the thermal and plasma CVD, respectively. The multiple SEM images were taken for each sample used for the plot in **g** and **h**. The Δ*S*_Ni_ was calculated as an averaged value obtained from those multiple SEM images. The range between maximum and minimum value of Δ*S*_Ni_ for each sample is shown with the error bars. (**i**,**j**) Concentration rates of carbon (black line) and Ni (red line) in Ni thin-film (70 nm) after (**i**) thermal CVD and (**j**) plasma CVD as a function of sputtering time (sputtering time is proportional to depth in Ni). Blue dashed line in **i** and **j** shows the carbon concentration of initial Ni film (untreated). (**k**) Comparison of (Σ*I*_C*1s*_/Σ*I*_Ni*2p2/3*_) values between thermal and plasma CVD.

Because systematic investigations were required to obtain the answers to these questions, we used a thin Ni film instead of the Ni nanobar. Note that the GNR growth from the thin Ni film was also confirmed to be similar to that with the Ni nanobar (Supplementary Fig. 7; Supplementary Note 6). To answer the former question, the stability of Ni is one of the key issues. To quantitatively investigate the stability of the Ni film, we measured the depression rate of the surface coverage of Ni (Δ*S*_Ni_) based on SEM images using automatic image processing (Supplementary Fig. 8; Supplementary Note 7). The Δ*S*_Ni_ values were analysed for various CVD experiments as functions of the CVD time, temperature and CVD type (thermal or plasma). Interestingly, a clear difference in the Δ*S*_Ni_ values could be observed between the thermal CVD and plasma CVD. In the case of the thermal CVD, Δ*S*_Ni_ reached around 80% at 750 °C (Fig. 5a–c,g), whereas Δ*S*_Ni_ could be maintained at a low value (below 20%) even at 900 °C (Fig. 5d–f,g) with plasma CVD. A similar difference was found for the growth-time dependence. The value of Δ*S*_Ni_ rapidly increased at around 5–10 min of growth with thermal CVD (800 °C; Fig. 5h), whereas the value of Δ*S*_Ni_ did not reach 10% even after 20 min with plasma CVD (800 °C) (Fig. 5h). To elucidate the most critical reason for this difference between thermal CVD and plasma CVD, the atomic components were analysed using X-ray photoelectron spectroscopy for the Ni film after thermal CVD and plasma CVD at 800 °C. The depth profile of the Ni film was carefully analysed in combination with Ar ion sputtering. In case of the Ni film after thermal CVD, the carbon concentration on the surface of the Ni was slightly higher than that of the untreated sample (Fig. 5i). However, the carbon concentration obviously decreased inside the Ni, which was at the same level as the untreated one. On the other hand, more than 10% of the carbon could be found inside the Ni after plasma CVD (Fig. 5j). The proportion of the integrated intensity of C*1s* to Ni*2p*^*2/3*^ (Σ*I*_*C1s*_/Σ*I*_*Ni2p2/3*_) was also calculated (Fig. 5k), where Sigma (Σ) represents the summation of peak intensity for C*1s* (*I*_*C1s*_) and Ni*2p*^*2/3*^ (*I*_*Ni2p2/3*_) along the film thickness direction. This indicated that the carbon concentration in the Ni after plasma CVD was at least three times higher than that after thermal CVD (Fig. 5k).

**Figure 6:**
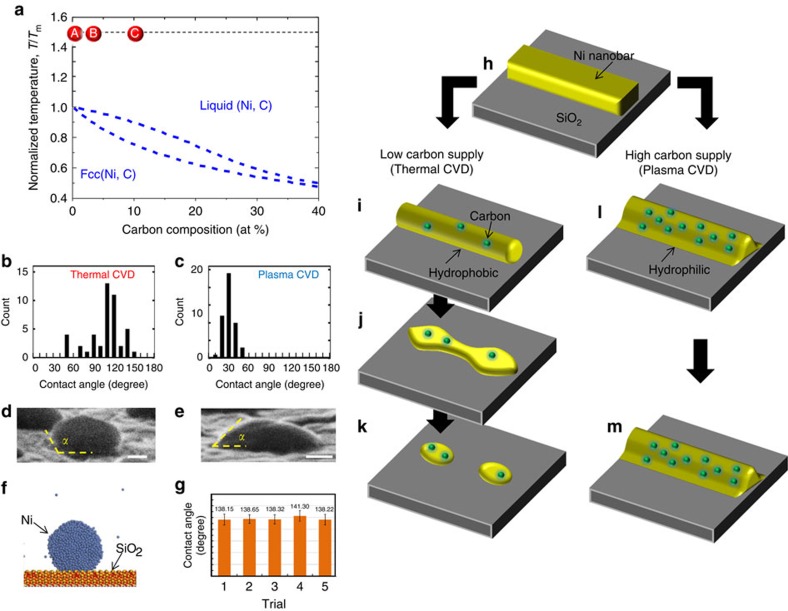
Model for Ni nanobar stability. (**a**) Typical phase diagram of Ni–C binary system. The points A,B and C in **a** correspond to the state of Ni nanobar during simple annealing with Ar, thermal CVD and plasma CVD, respectively. (**b**–**e**) (**b**,**c**) Histogram of contact angle and (**d**,**e**) typical SEM images for Ni+C nanoparticles formed by (**b**,**d**) thermal CVD and (**c**,**e**) plasma CVD, respectively. Scale bar, 200 nm (**d**,**e**). (**f**) Results of the molecular dynamics simulation for Ni nanoparticles on SiO_2_. (**g**) Contact angle of Ni nanoparticle on a SiO_2_ substrate given by five replicate molecular dynamics simulations. The maximum and minimum value of each error bars in **g** corresponds to the maximum and minimum contact angle over the calculation time (from 50 to 100 ps). (**h**–**m**) Schematic illustration of Ni nanobar (**h**) before and after (**i**–**k**) thermal CVD and (**l**,**m**) plasma CVD.

The origin of the differences in the thermal stability of the Ni film between thermal CVD and plasma CVD could be explained with the phase diagram of the Ni–C system by considering the carbon-concentration differences (see Methods and Supplementary Fig. 9; Supplementary Note 8). In general, there are three main phases in the Ni–C phase diagram: liquid (Ni+C), face-centred cubic (fcc) solid solution (Ni+C), and graphite. To discuss the stability of Ni, we have to focus on the phase equilibrium between the liquid and fcc under the high-temperature condition (before the cooling stage). Because the graphene should be formed during the cooling process, we assumed that the graphitization of solid carbon did not occur under the high-temperature (900 °C) condition. On the basis of this assumption, the phase diagram could be drawn as shown in Fig. 6a. The melting point of a nanoscale material is sensitive to its structure and known to decrease by several hundred degrees from that of the bulk crystal^45^. In this study, it was confirmed that the melting point of the Ni nanobar was between 550 and 600 °C (Supplementary Fig. 10; Supplementary Note 9). Therefore, to make an appropriate comparison between the results of the calculation and experiment with Ni nanobar, we used a temperature normalized by the melting point of the Ni (bulk crystal or nanobar) (*T*_m_) (*T*/*T*_m_) instead of the absolute one. The two blue lines in Fig. 6a denote the phase boundaries between the liquid and fcc phases. As a control experiment, we first performed simple annealing of Ni nanobar under the Ar atmosphere (250 Pa) without CH_4_ at 900 °C for the same process time as the CVD growth. After the simple annealing, the Ni nanobar was converted to Ni nanoparticles, indicating that the Ni nanobar should be in the liquid phase at 900 °C (Supplementary Fig. 10). Thus, we plotted the state of the Ni nanobar during simple annealing at 900 °C as point A in Fig. 6a, where *T*/*T*_m_=1.5 (we assumed the melting point of Ni nanobar is 600 °C due to the nanoscale effect as shown in Supplementary Fig. 10). For the Ni nanobar during the thermal CVD, the temperature was the same (900 °C), but carbon was weakly supplied from the CH_4_ gas phase. Thus, it could be plotted at position point B in Fig. 6a. Because the Ni nanobar in the plasma CVD included a higher concentration (∼10%) of carbon, it was possible to plot it at position point C in Fig. 6a. Note that the liquid temperature gradually decreases with increasing carbon composition in Fig. 6a. Hence, these indicate that all three states (point A, point B and point C in Fig. 6a) should be the liquid phase of Ni+C under the high-temperature condition (900 °C).

To obtain the answer for the first question, we have to discuss the following point about why the stability of the Ni+C liquid phase varies between the thermal and plasma CVD at 900 °C. This can be explained by the difference in the wetting probability of the Ni+C liquid on a SiO_2_ substrate. Figs 6b,c and 6d,e shows histograms of the contact angle and typical SEM images, respectively, of a Ni nanoparticle after thermal CVD (Fig. 6b,d) and plasma CVD (Fig. 6c,e). Note that a slightly higher temperature and longer process time were used to enhance the morphology transition of the Ni thin-film to nanoparticles, which was required to measure the contact angle. In case of the thermal CVD, almost all of the Ni+C nanoparticles showed larger contact angles, with an average value of 120° (Fig. 6b,d), whereas the plasma treated Ni+C nanoparticles tended to show smaller contact angles, with an average of 30° (Fig. 6c,e). This indicated that the thermal and plasma treated Ni+C particles were hydrophobic-like and hydrophilic-like state, respectively (Supplementary Fig. 11; Supplementary Note 10). To support this experimental result, the wettability of the Ni nanoparticles on SiO_2_ was also estimated using a molecular dynamics simulation, revealing that the pure Ni nanoparticles tended to have higher contact angles on a SiO_2_ substrate, with an average angle of 138.9° and a hydrophobic-like behaviour (Fig. 6f,g; see Methods section, Supplementary Figs 12–14, and Supplementary Note 11 for more details). This is consistent with the hydrophobic-like behaviour of Ni+C nanoparticles with a lower carbon concentration (thermal CVD). The hydrophobic-like nanobar tended to form particle-like shapes to satisfy the minimum surface energy, as explained by the Plateau–Rayleigh (P–R) instability^46^. Hence, during thermal CVD the Ni nanobar was in a hydrophobic-like liquid state as a result of the lower carbon concentration, resulting in a morphology change from the nanobar to nanoparticles (Fig. 6h–k). In contrast, in the plasma CVD, a higher concentration of carbon could be supplied to the Ni nanobar, forming a hydrophilic-like liquid state, which was relatively stabler than a hydrophobic-like liquid, making it possible to maintain the Ni nanobar structure at a high-temperature condition (Fig. 6h,l,m). The decrease of contact angle of Ni+C liquid with carbon supply can be explained by considering the surface tension of Ni+C liquid. The contact angle (*α*) can be calculated with the Young equation: cos *α*=(*γ*_S_−*γ*_LS_)/γ_L_, where *γ*_S_, *γ*_L_, *γ*_LS_ shows surface tension of solid (SiO_2_), liquid (Ni+C) and solid (SiO_2_)-liquid (Ni+C) interface, respectively. On the basis of the literatures, it was reported that the surface tension of metal liquid can decrease with an increase in carbon concentration^47^. The low *γ*_L_ increases cos *α*, resulting in the low contact angle. This address the question about why the Ni nanobar was stable in the plasma CVD even under a high-temperature condition.

**Figure 7:**
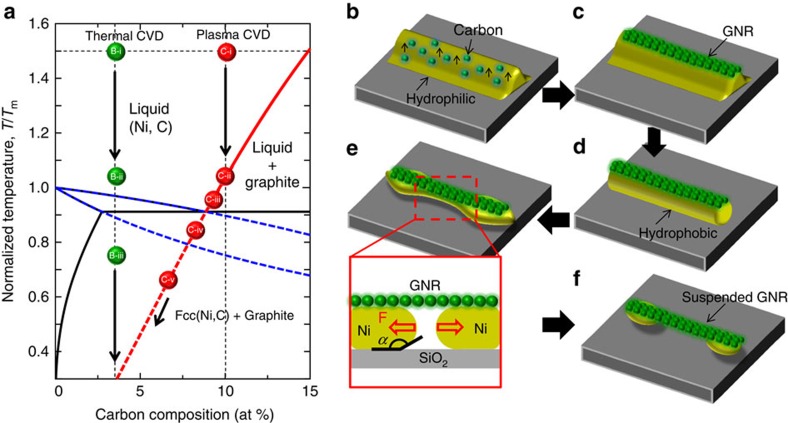
Formation model of suspended GNRs. (**a**) Typical phase diagram of Ni–C binary system. (**b**–**f**) Schematic illustration of suspended GNR nucleation process. Each process has the following steps, (**b**) just before cooling, (**c**,**d**) GNR segregation at initial cooling process, (**e**) dewetting of Ni nanoparticle under GNR and (**f**) suspended GNR. Each step of **b**,**c**,**d**,**e** and **f** corresponds to the position of point C-i, C-ii, C-iii, C-iv and C-v in **a**, respectively. Inset in **e** is the schematic cross sectional image of Ni nanobar just after the breaking due to P–R instability.

Next, we will discuss the second issue, which is about how the suspended GNRs can be formed during the cooling process. Because graphitic crystallization should occur during the cooling process as a result of the non-isothermal reaction, another phase diagram was calculated by considering the following three states: liquid, fcc, and graphite (Fig. 7a; see Methods section and Supplementary Fig. 9b). As discussed in Fig. 6a, the starting point during the cooling process for thermal and plasma CVD can be plotted point B-i and point C-i in Fig. 7a, respectively. The representative positions of step by step cooling process for point B and point C are described with i to iii and i to v, respectively (Fig. 7a). By decreasing the temperature, point B-i and point C-i can move to the low-temperature position with keeping initial carbon concentration. Once it crosses to the red curve in Fig. 7a, graphitic crystallization should occur. In case of thermal CVD, it cannot cross to the red curve until very low temperature (*T*/*T*_m_=0.3), indicating GNR growth is not possible with thermal CVD. The states of Ni nanobar at point B-i, point B-ii and point B-iii in Fig. 7a correspond with that shown in Fig. 6i–k, respectively. For the plasma CVD, it can cross to the red curve around the high-temperature condition (*T*/*T*_m_=1.05), then move to the low-temperature side by following the red curve. At this stage, the GNRs could be nucleated on the surface of the liquid (point C-ii and point C-iii in Fig. 7a, Fig. 7b,c). In this case, a large number of carbon atoms were used to form GNRs (Fig. 7b), and the carbon concentration in the liquid rapidly decreased (Fig. 7c), which could cause the change in the liquid state from hydrophilic-like to hydrophobic-like, as previously discussed (Fig. 7d). Then, the Ni+C liquid under the GNRs became unstable and tended to form particle-like structures due to the P–R instability (Fig. 7e). It should be mentioned that there is another possibility causing the Ni nanobar breaking, which is end pinching. Then, further detailed explanation of the breaking of Ni nanobar liquid was considered. It was reported that the breaking of continuous fluid is sensitive to the aspect ratio of fluid 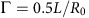 and Ohnesorge number 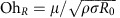, where *L*, *R*_0_, *μ*, *ρ* and *σ*, denotes length, radius, dynamic viscosity, density and surface tension of liquid, respectively^48^. The simple calculation shows that the Oh_*R*_ for Ni nanobar (20-50 nm width (*R*_0_=10–25 nm)) is Oh_*R*_=0.215–0.34. It was reported that there is a threshold for Oh_*R*,_ around 0.1, and lower and higher Oh_*R*_ than 0.1 causes end pinching and P–R instability, respectively. Thus, the breaking of Ni nanobar liquid in our experiment should be caused by not end pinching, but P–R instability. In this case, the aspect ratio Γ=10–25 (*L*=500 nm) matches with the unstable fluid region in Γ-Oh_*R*_ plot in ref. 48 (Supplementary Fig. 15; Supplementary Note 12), showing the Ni nanobar liquid can be broken by P–R instability. The wavelength of perturbation (*λ*) can be also written by the simple correlation *λ*≃9.02*R*_0_ (ref. 49). To confirm the possibility of P–R instability, we also counted the GNR length grown from 20 nm width (*R*_0_=10 nm) of Ni nanobar. Interestingly, almost all of GNRs has longer than 80–100 nm, which is almost same with the *λ*≃90.2 nm (Supplementary Fig. 16; Supplementary Note 12). This also indicates that the P–R instability should be one of the possible explanations causing the breaking of liquid-phase Ni nanobar.

After the breaking of Ni+C nanobar liquid, it received a capillary force (*F*), which is defined by the following equation^50^.


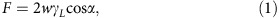


where *w* denote the GNR width. When we assumed that the width of the Ni nanobar and GNR was 50 nm, and *α*=120°, the equation (1) gives *F*=88.5 nN (we used the value of γ_L_ for pure Ni in the liquid phase at 1,728 K (melting point), which is 1.77 Nm^−1^ (ref. 51)). Since *α* is obviously larger than 90° as shown in Fig. 6b,d,f,g the direction of *F* is towards the side of the GNR (Fig. 7e). The relatively high surface energy of Ni+C nanobar liquid also causes the morphology change to particle-like shapes of Ni+C liquid to satisfy the minimum surface energy. Simple calculation shows that the surface to volume ratio of Ni+C nanobar decreases with length of Ni+C nanobar, reaching 64% (length: 60 nm) of initial structures (length: 250 nm; Supplementary Fig. 17; Supplementary Note 13). On the basis of these results, it can be revealed that the combination of capillary force towards GNR end and morphology change caused by surface-energy stability can cause the movement of Ni+C liquid under GNR to the end of GNR, resulting in the formation of suspended GNRs (Fig. 7f). This can be the answer of the second question about the formation mechanism of suspended GNRs.

## Discussion

As previously discussed, through the comparison of experimental results, molecular dynamics simulations and theoretical calculations with the phase-diagram theory, we established a possible growth model for suspended GNRs from Ni nanobars. Since the segregation of GNRs happened within very short period during the cooling process, we think that the non-equilibrium reaction should mainly govern the formation process of GNR in our method. The overall tendency of experimental results can be well explained by traditional phase diagram, which is based on the equilibrium state. This knowledge should be useful not only for GNR synthesis but also for the synthesis of other types of nanomaterials with high controllability. It is also true that further progress is required to have the quantitative discussion for the growth model of GNRs, which can be realized with advanced phase-diagram analysis considering the non-equilibrium state and also nanoscale effects. This can be the future work of this study. Recently, a similar crystal growth with P–R instability has been reported for nanowire synthesis^52^, indicating that these unique reactions have huge potential as a future method for nanomaterial synthesis with the desired controllability.

In summary, the high-yield (over 98%) synthesis of over 1,000,000 suspended GNR arrays in a wafer-scale (2 × 2 cm^2^) was realized using a RH-PCVD method. Polarized Raman analysis revealed that the edge structure of suspended GNRs arrays can have very uniform structure with near zigzag orientation dominant. A locally addressable side-gate transistor operation and highly conductive short-channel structures could also be demonstrated with the suspended GNRs. Furthermore, the detailed growth dynamics of suspended GNRs were also investigated based on systematic experiments, molecular dynamics simulations and theoretical calculations. The high rate of carbon supply during plasma CVD was one of the critical factors to stabilize the liquid-phase Ni nanobar under the high-temperature condition because of the wettability change from hydrophobic-like to hydrophilic-like state. After the nucleation of GNRs during the cooling process, the P–R instability could break nanobars into nanoparticles, and the combination of capillary force and surface-energy balance could move the liquid-phase Ni into both ends of the GNR, forming a suspended GNR shape.

## Methods

### Plasma CVD

A homemade plasma CVD system was used for the RH-PCVD. Before the plasma CVD growth, an electric furnace was heated to the desired temperature (typically 800–900 °C) under flowing hydrogen (50 Pa). A substrate was immediately transferred to the center area, and rapid heating was performed. CH_4_ and H_2_ gases at a 9:1 flow ratio (250 Pa) were flowed just after reaching a fixed heating time (typically 60 s) where the Ni nanobar was not dissipated. After that, the radio frequency power (60 W, 13.56 MHz) was supplied to the coils outside of the quartz tube. The plasma irradiation time was typically 5–30 s. Following the plasma CVD, the substrate was moved from the center to the outside of the electrical furnace to rapidly decrease the temperature of the substrate.

### Characterizations

The structure of the GNR sample was characterized using SEM (JEOL JSM-7100F and Hitachi SU1510, Japan). The electrical measurements of the GNR devices were performed using a vacuum-probe station with a semiconductor parameter analyser (HP 4155 C). The elementary analysis of the Ni film was performed using X-ray photoelectron spectroscopy (Ulvac-phi, ESCA1600, Japan). The polarized Raman measurements (HR-800, Horiba) were carried out using the VV configuration, where the orientation of the polarizer for inspecting the scattered light was parallel to the polarization of the incident light. An Ar laser with a 488-nm wavelength was used for the excitation. The mappings were performed with 200–500 nm steps.

### Phase-diagram calculations

The phase diagram of the Ni–C binary system was calculated using the CALPHAD method with the thermodynamic parameters reported by Guillermet^53^. The regular solution approximation was applied to the liquid phase, while the Ni-rich fcc phase was moulded using a two-sublattice model. No solubility was taken into account for the thermodynamic state of graphite. The phase diagram was constructed from these thermodynamic models based on the common tangent rule.

### Molecular dynamics simulations

The wettability of a Ni nanoparticle on a SiO_2_ substrate was examined with a classical molecular dynamics simulation using MS ForcitePlus in Materials Studio 8.0 (ref. 54). The Condensed Phase Optimized Molecular Potentials for Atomic Simulation Studies II (COMPASS II) was employed for the interatomic potential^55^,^56^. The cutoff distance for short-range interactions was set to 12.5 Å. The Coulomb interactions were calculated using the Ewald method. The velocity Verlet algorithm was used to integrate the classical equation of motion with a time step of 2.0 fs. A Nose thermostat was applied to control the temperature.

### Data availability

The authors declare that the data supporting the findings of this study are available within the article and its Supplementary Information files.

## Additional information

**How to cite this article:** Suzuki, H. *et al*. Wafer-scale fabrication and growth dynamics of suspended graphene nanoribbon arrays. *Nat. Commun.* 7:11797 doi: 10.1038/ncomms11797 (2016).

## Supplementary Material

Supplementary Information Supplementary Figures 1-17, Supplementary Notes 1-13 and Supplementary References

## References

[b1] Novoselov K. S. . Electric field effect in atomically thin carbon films. Science 306, 666–669 (2004). 15499015 10.1126/science.1102896

[b2] Geim A. K. & Novoselov K. S. The rise of graphene. Nat. Mater. 6, 183–191 (2007). 17330084 10.1038/nmat1849

[b3] Splendiani A. . Emerging photoluminescence in monolayer MoS_2_. Nano Lett. 10, 1271–1275 (2012). 20229981 10.1021/nl903868w

[b4] Radisavljevic B., Radenovic A., Brivio J., Giacometti V. & Kis A. Single-layer MoS_2_ transistors. Nat. Nanotechnol. 6, 147–150 (2011). 21278752 10.1038/nnano.2010.279

[b5] Lin Y.-M. . 100-GHz transistors from wafer-scale epitaxial graphene. Science 327, 662 (2010). 20133565 10.1126/science.1184289

[b6] Novoselov K. S. . Room-temperature Quantum Hall effect in graphene. Science 315, 1379 (2007). 17303717 10.1126/science.1137201

[b7] Mak K. F., He K., Shan J. & Heinz T. F. Control of valley polarization in monolayer MoS_2_ by optical helicity. Nat. Nanotechnol. 7, 494–498 (2012). 22706698 10.1038/nnano.2012.96

[b8] Lee C. . Measurement of the elastic properties and intrinsic strength of monolayer graphene. Science 321, 385–388 (2008). 18635798 10.1126/science.1157996

[b9] Lee J. . High frequency MoS_2_ nanomechanical resonators. ACS Nano 7, 6086–6091 (2013). 23738924 10.1021/nn4018872

[b10] Cao J., Wang Q. & Dai H. Electron transport in very clean, as-grown suspended carbon nanotubes. Nat. Mater. 4, 745–749 (2005). 16142240 10.1038/nmat1478

[b11] Lefebvre J., Homma Y. & Finnie P. Bright band gap photoluminescence from unprocessed single-walled carbon nanotubes. Phys. Rev. Lett. 90, 217401 (2003). 12786586 10.1103/PhysRevLett.90.217401

[b12] Du X., Skachko I., Barker A. & Andrei E. Y. Approaching ballistic transport in suspended graphene. Nat. Nanotechnol. 3, 491–495 (2008). 18685637 10.1038/nnano.2008.199

[b13] Chen C. . Graphene mechanical oscillators with tunable frequency. Nat. Nanotechnol. 8, 923–927 (2013). 24240431 10.1038/nnano.2013.232

[b14] Sun Y. & Rogers J. A. Fabricating semiconductor nano/microwires and transfer printing ordered arrays of them onto plastic substrates. Nano Lett. 4, 1953–1959 (2004).

[b15] Shi H. . Exciton dynamics in suspended monolayer and few-layer MoS_2_ 2D crystals. ACS Nano 7, 1072–1080 (2013). 23273148 10.1021/nn303973r

[b16] Wu S. . Control of two-dimensional excitonic light emission via photonic crystal. 2D Mater. 1, 011001 (2014).

[b17] Verhagen E., Deléglise S., Weis S., Schliesser A. & Kippenberg T. J. Quantum-coherent coupling of a mechanical oscillator to an optical cavity mode. Nature 482, 63–67 (2012). 22297970 10.1038/nature10787

[b18] Mann D. . Electrically driven thermal light emission from individual single-walled carbon nanotubes. Nat. Nanotechnol. 2, 33–38 (2007). 18654204 10.1038/nnano.2006.169

[b19] Rickhaus P. . Ballistic interferences in suspended graphene. Nat. Commun. 4, 2342 (2013). 23946010 10.1038/ncomms3342

[b20] Chen C. . Performance of monolayer graphene nanomechanical resonators with electrical readout. Nat. Nanotechnol. 4, 861–867 (2009). 19893525 10.1038/nnano.2009.267

[b21] Han M. Y., Ozyilmaz B., Zhang Y. B. & Kim P. Energy band-gap engineering of graphene nanoribbons. Phys. Rev. Lett. 98, 206805 (2007). 17677729 10.1103/PhysRevLett.98.206805

[b22] Li X. L., Wang X. R., Zhang L., Lee S. W. & Dai H. J. Chemically derived, ultrasmooth graphene nanoribbon semiconductors. Science 319, 1229–1232 (2008). 18218865 10.1126/science.1150878

[b23] Nakada K., Fujita M., Dresselhaus G. & Dresselhaus M. S. Edge state in graphene ribbons: nanometer size effect and edge shape dependence. Phys. Rev. B 54, 17954–17961 (1996). 10.1103/physrevb.54.17954 9985930

[b24] Wakabayashi K., Fujita M., Ajiki H. & Sigrist M. Electronic and magnetic properties of nanographite ribbons. Phys. Rev. B 59, 8271–8282 (1999).

[b25] Chen Z. H., Lin Y. M., Rooks M. J. & Avouris P. Graphene nano-ribbon electronics. Physica E 40, 228–232 (2007).

[b26] Pan Z. . Wrinkle engineering: a new approach to massive graphene nanoribbon arrays. J. Am. Chem. Soc. 133, 17578–17581 (2011). 21981554 10.1021/ja207517u

[b27] Jiao L. Y., Zhang L., Wang X. R., Diankov G. & Dai H. J. Narrow graphene nanoribbons from carbon nanotubes. Nature 458, 877–880 (2009). 19370031 10.1038/nature07919

[b28] Kosynkin D. V. . Longitudinal unzipping of carbon nanotubes to form graphene nanoribbons. Nature 458, 872–876 (2009). 19370030 10.1038/nature07872

[b29] Wang X. . Graphene nanoribbons with smooth edges behave as quantum wires. Nat. Nanotechnol. 6, 563–567 (2011). 21873992 10.1038/nnano.2011.138

[b30] Sprinkle M. . Scalable templated growth of graphene nanoribbons on SiC. Nat. Nanotechnol. 5, 727–731 (2010). 20890273 10.1038/nnano.2010.192

[b31] Baringhaus J. . Exceptional ballistic transport in epitaxial graphene nanoribbons. Nature 506, 349–354 (2014). 24499819 10.1038/nature12952

[b32] Cai J. . Atomically precise bottom-up fabrication of graphene nanoribbons. Nature 466, 470–473 (2010). 20651687 10.1038/nature09211

[b33] Cai J. . Graphene nanoribbon heterojunctions. Nat. Nanotechnol. 9, 896–900 (2014). 25194948 10.1038/nnano.2014.184

[b34] Lin M.-W. . Room-temperature high on/off ratio in suspended graphene nanoribbon field-effect transistors. Nanotechnol 22, 265201 (2011). 10.1088/0957-4484/22/26/265201 21576804

[b35] Kato T. & Hatakeyama R. Site- and alignment-controlled growth of graphene nanoribbons from nickel nanobars. Nat. Nanotechnol. 7, 651–656 (2012). 22961304 10.1038/nnano.2012.145

[b36] Ruffieux P. . On-surface synthesis of graphene nanoribbons with zigzag edge topology. Nature 1511, 05037 (2016). 10.1038/nature17151 27008967

[b37] Jacobberger R. M. . Direct oriented growth of armchair graphene nanoribbons on germanium. Nat. Commun. 6, 8006 (2015). 26258594 10.1038/ncomms9006 PMC4918381

[b38] Xie L. . Graphene nanoribbons from unzipped carbon nanotubes: atomic structures, Raman spectroscopy, and electrical properties. J. Am. Chem. Soc. 133, 10394–10397 (2011). 21678963 10.1021/ja203860a

[b39] Jia X. . Controlled formation of sharp zigzag and armchair edges in graphitic nanoribbons. Science 323, 1701–1705 (2009). 19325109 10.1126/science.1166862

[b40] Girit C. Ö. . Graphene at the edge: stability and dynamics. Science 323, 1705–1708 (2009). 19325110 10.1126/science.1166999

[b41] Krauss B. . Raman scattering at pure graphene zigzag edges. Nano Lett. 10, 4544–4548 (2010). 20945848 10.1021/nl102526s

[b42] Li X. . Large-area synthesis of high-quality and uniform graphene films on copper foils. Science 324, 1312–1314 (2009). 19423775 10.1126/science.1171245

[b43] Li X., Cai W., Colombo L. & Ruoff R. S. Evolution of graphene growth on Ni and Cu by carbon isotope labeling. Nano Lett. 9, 4268–4272 (2009). 19711970 10.1021/nl902515k

[b44] Weatherup R. S., Dlubak B. & Hofmann S. Kinetic control of catalytic CVD for high-quality graphene at low temperatures. ACS Nano 6, 9996–10003 (2012). 23025628 10.1021/nn303674g

[b45] Engelmann Y., Bogaerts A. & Neyts E. C. Thermodynamics at the nanoscale: phase diagrams of nickel–carbon nanoclusters and equilibrium constants for phase transitions. Nanoscale 6, 11981–11987 (2014). 25177915 10.1039/c4nr02354d

[b46] Plateau J. A. F. Experimental and theoretical researches on the figures of equilibrium of a liquid mass withdrawn from the action of gravity The Annual Report of the Board of Regents of the Smithsonian Institution, 270–285Gover Printing Office, Washington, DC (1863).

[b47] Deyev G. F. Surface Phenomena in Fusion Welding Processes CRC Press (2005).

[b48] Driessen T. . Stability of viscous long liquid filaments. Phys. Fluids 25, 062109 (2013).

[b49] Bush J. W. M. MIT Lecture Notes on Surface Tension lecture 5 Massachusetts Institute of Technology (2004) Available at http://www.google.co.jp/url?sa=t&rct=j&q=&esrc=s&source=web&cd=1&cad=rja&uact=8&ved=0ahUKEwjo0-mr45fMAhVEraYKHdC3BioQFggbMAA&url=http%3A%2F%2Fweb.mit.edu %2F2.21%2Fwww%2FLec-notes %2FSurfacetension%2FLecture5.pdf&usg=AFQjCNGGd0OR0FgjyEJOHsyB1NJiTNksDg &sig2=Bmttp-cTFEWNx7nQECICvw.

[b50] Pozrikidis C. Introduction to Theoretical and Computational Fluid Dynamics Oxford Univ. Press (2011).

[b51] Brillo J. & Egry I. Surface tension of nickel, copper, iron and their binary alloys. J. Mater. Sci. 40, 2213–2216 (2005).

[b52] Day R. W. . Plateau–Rayleigh crystal growth of periodic shells on one-dimensional substrates. Nat. Nanotechnol. 10, 345–352 (2015). 25751303 10.1038/nnano.2015.23

[b53] Guillermet A. F. Thermodynamic properties of the Fe-Co-Ni-C system. Z. Metallkd. 79, 524–536 (1988).

[b54] BIOVIA Materials Studio 8.0, Dassault Systemes Biovia Cambridge, UK (2015).

[b55] Sun H. COMPASS: an ab initio force-field optimized for condensed-phase applications—overview with details on alkane and benzene compounds. J. Phys. Chem. B 102, 7338–7364 (1998).

[b56] Sun H. & Rigby D. Polysiloxanes: ab initio force field and structural, conformational and thermophysical properties. Spectrochim. Acta Part A 53, 1301–1323 (1997).

