# Supplementary material for: Wafer-scale fabrication and growth dynamics of suspended graphene nanoribbon arrays
Source: Nat Commun. 2016 Jun 2;7:11797. doi: 10.1038/ncomms11797 (PMC4895714; doi:10.1038/ncomms11797)
Supplement: Supplementary Information — Supplementary Figures 1-17, Supplementary Notes 1-13 and Supplementary References [file ncomms11797-s1.pdf]

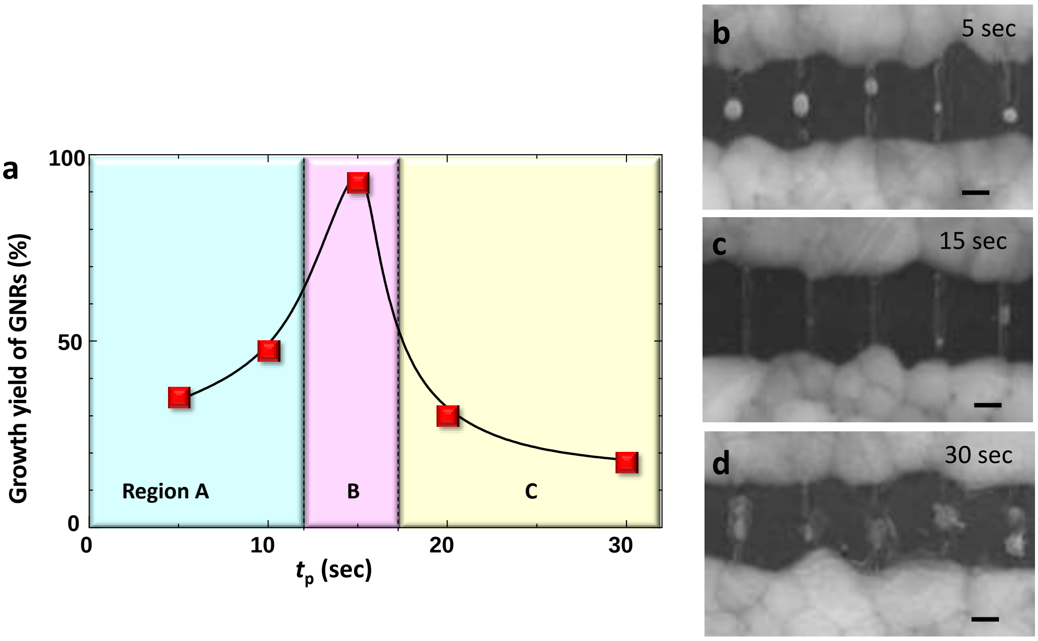

Supplementary Figure 1| Adjustment of growth conditions for GNR growth. (**a**) Growth yield of GNRs as a function of t_p. (**b-d**) Typical SEM image of product after the different t_p ((**b**) 5 sec, (**c**) 15 sec, (**d**) 30 sec). The scale bars in **b-d** show 100 nm.

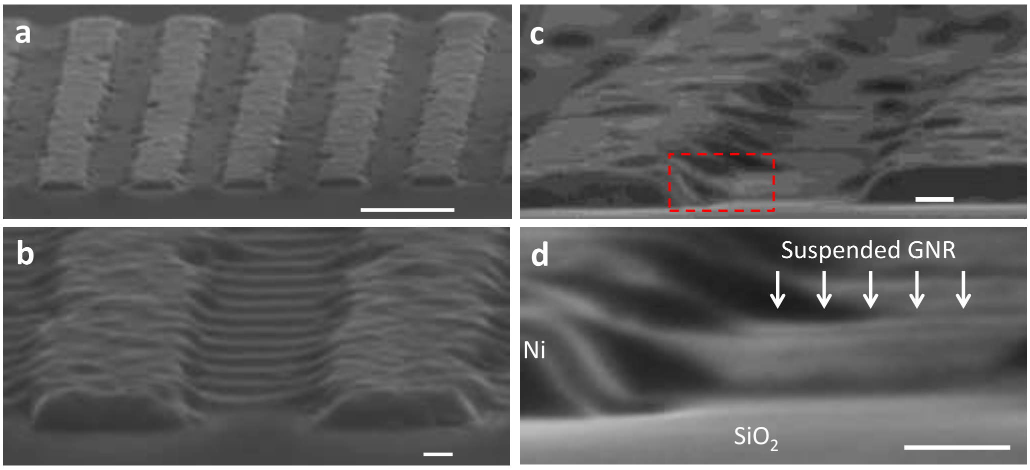

Supplementary Figure 2| Suspended structures of GNRs. Typical (**a**) low (**b,c**) middle, and (**d**) high magnification SEM images of suspended GNRs. The observed area in **d** is the same position of dashed frame in **c**. Arrows in **d** is the highlight of the suspended GNR. The scale bars in **a** and **b-d** are 1 μm and 100 nm, respectively.

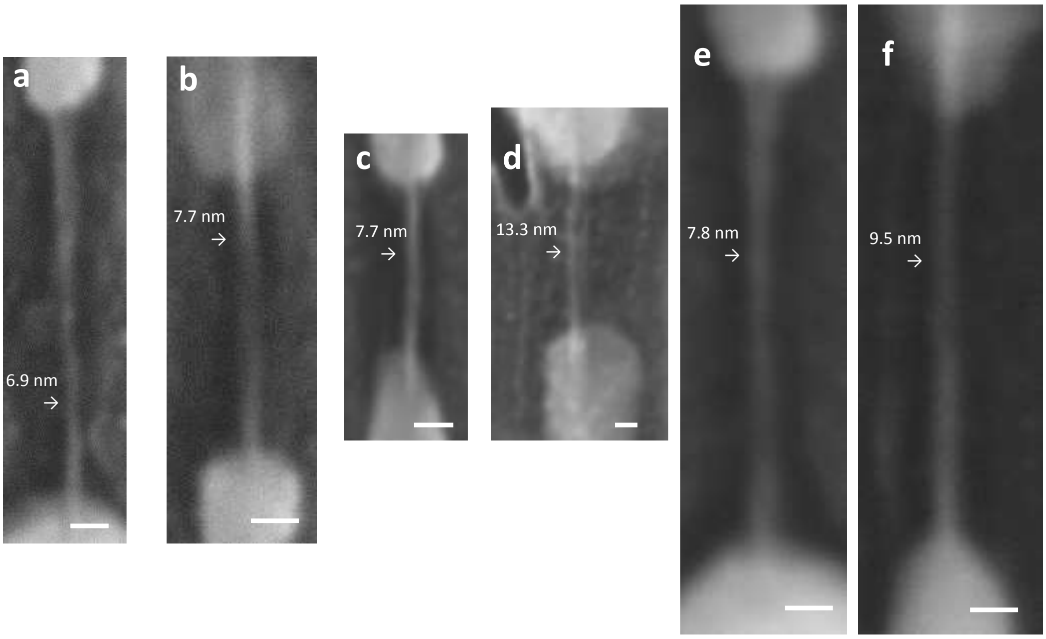

Supplementary Figure 3| Typical SEM images of sub 10 nm narrow GNRs. The smallest part of GNR width for (**a-f**) is 6.9 nm, 7.7 nm, 7.7 nm, 13.3 nm, 7.8 nm, 9.5 nm, respectively. The scale bars in **a-f** show 20 nm.

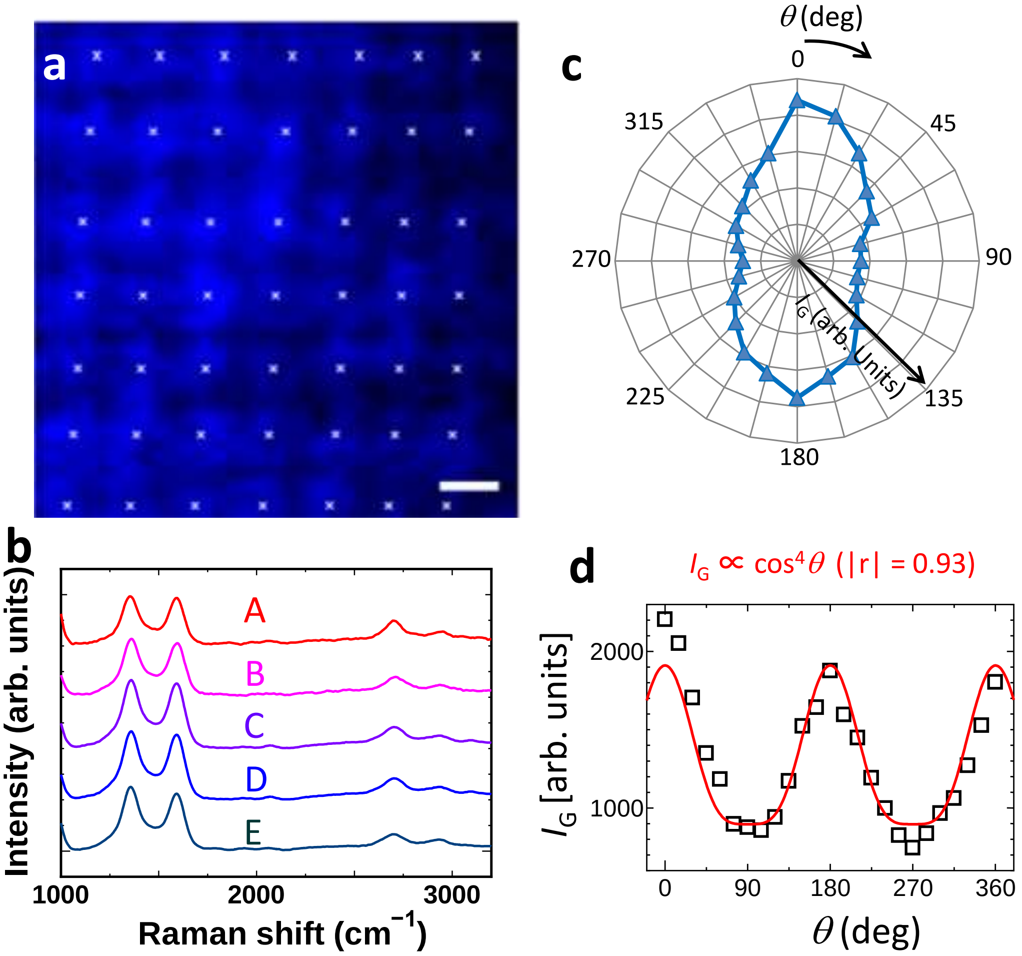

Supplementary Figure 4| Polarized G-band Raman features. (**a**) G-band Raman intensity mapping, (**b**) Raw Raman scattering spectra taken at different 5 points, (**c**) polar plot, and (**d**) intensity vs. θ plot of G-band in suspended GNRs. The scale bar in **a** shows 1 μm. The sign $\times$ in **a** corresponds to the position of each GNR.

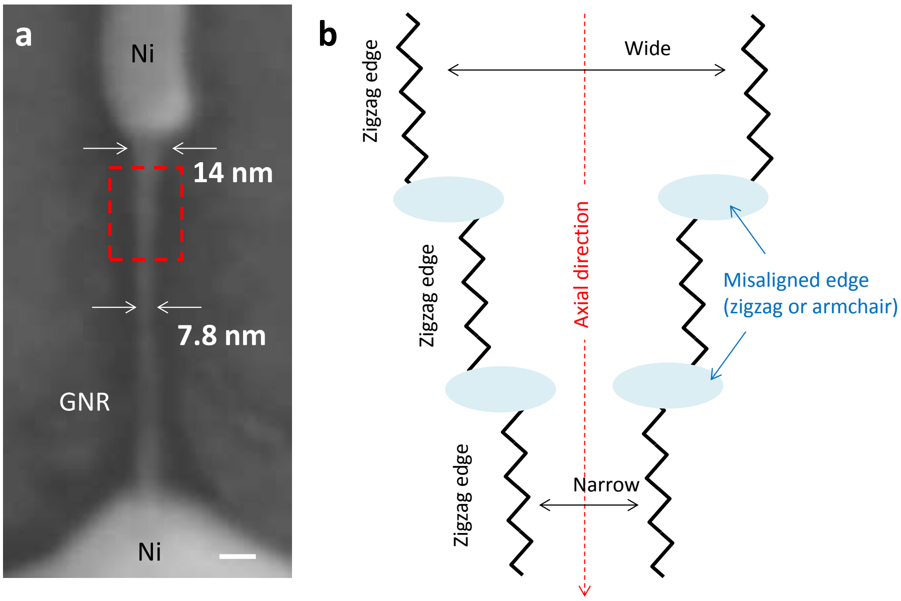

Supplementary Figure 5| Edge orientation model. (**a**) Typical SEM image of suspended GNR. The scale bar in **a** shows 20 nm. (**b**) Schematic illustration of aligned zigzag edge and misaligned edge in same GNR (square region in **a**). The red dotted arrow and black solid arrows in **b** show the direction parallel and perpendicular to the GNR axis, respectively.

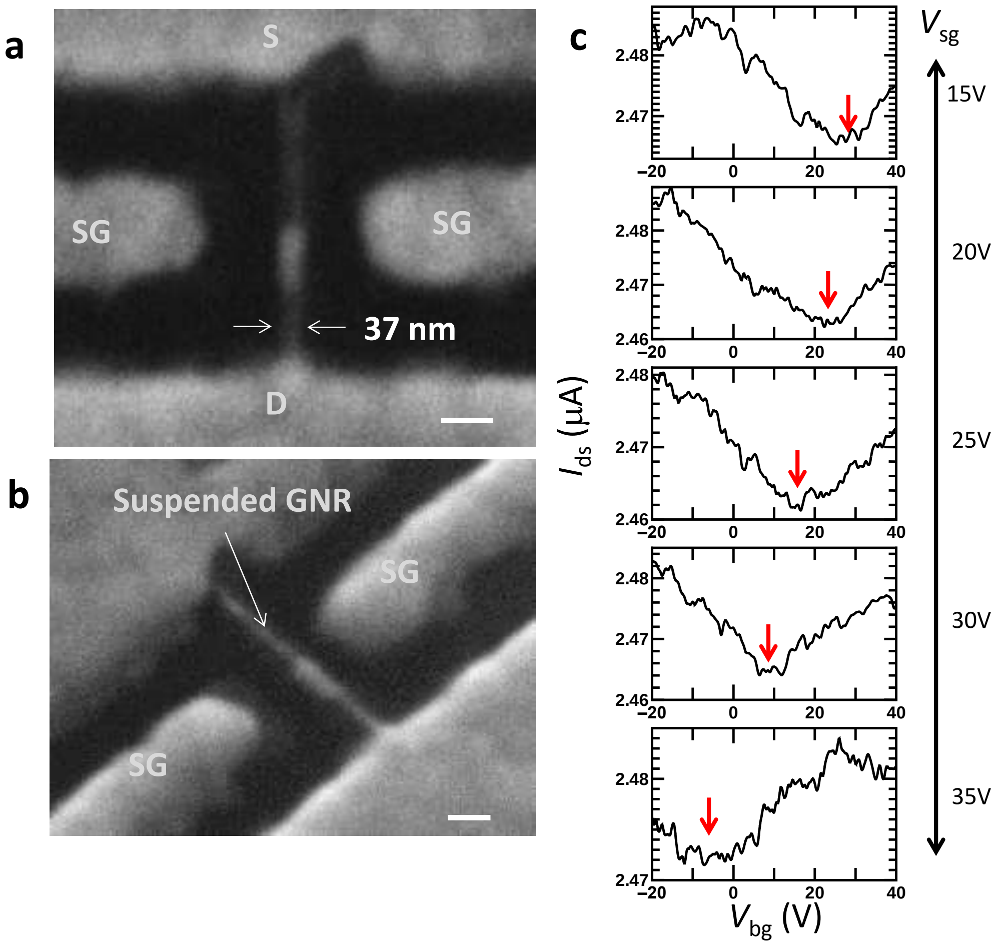

Supplementary Figure 6| Suspended GNR device with side gate operation. (**a**,**b**) SEM images of suspended GNR with side gate structures. The scale bars in **a**,**b** show both 100 nm. (**c**) I_{ds}–V_{bg} curves of suspended GNR under various V_{sg} values (top: 15 V to bottom: 35 V). The arrows in **c** denote the charge neutral point for each curve.

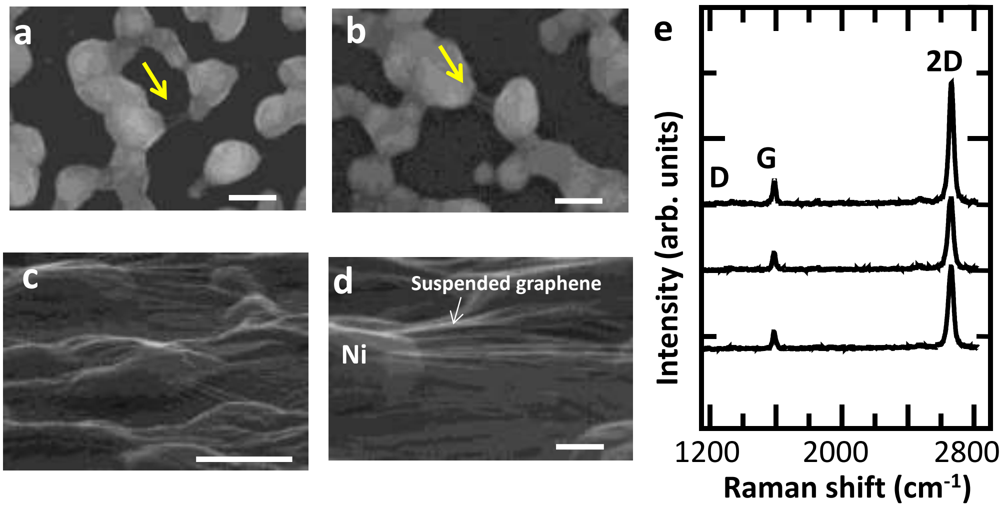

Supplementary Figure 7| Suspended GNRs growth with thin Ni film. (**a-d**) SEM images of (**a,b**) suspended GNR and (**c,d**) graphene grown from thin Ni film. The scale bars in **a,b,d** and **c** show 250 nm and 1 μm, respectively. (**e**) Typical Raman scattering spectra of suspended graphene grown from thin Ni film.

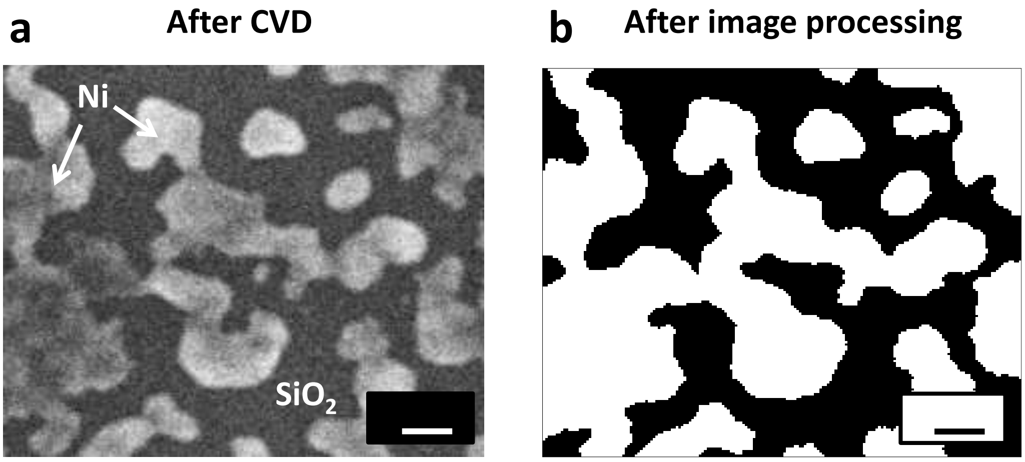

Supplementary Figure 8| Analysis of Ni film deformation. (**a**) SEM image of Ni film after CVD. (**b**) SEM image of Ni film after image processing. The scale bars in **a,b** show both 500 nm.

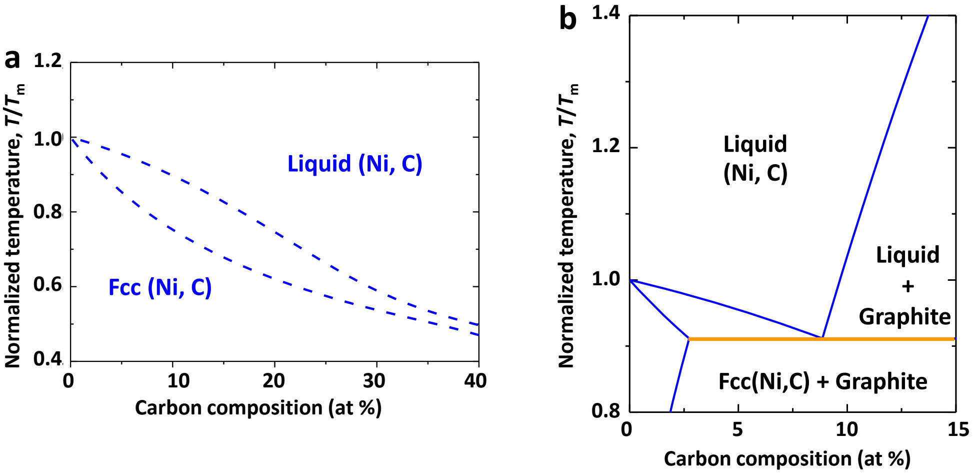

Supplementary Figure 9| Phase diagram analysis. (**a**) Phase equilibria between liquid and fcc, calculated by suspending precipitation of graphite. (**b**) The calculated phase diagram near the Ni-rich part.

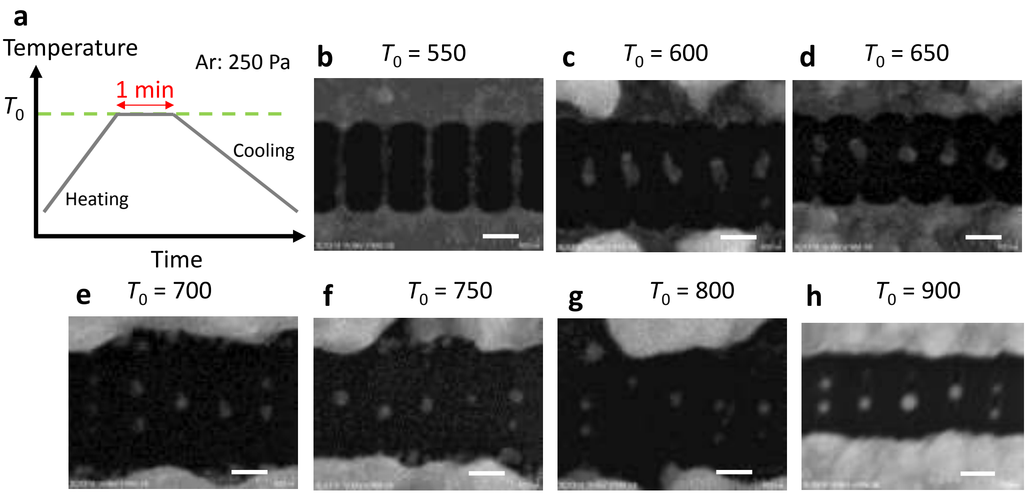

Supplementary Figure 10| Stability of Ni nanobar without carbon supply. (**a**) Time sequence of annealing experiment. (**b-h**) Typical SEM images of Ni nanobar after the annealing at different temperature conditions. The scale bars in **b-h** show 200 nm.

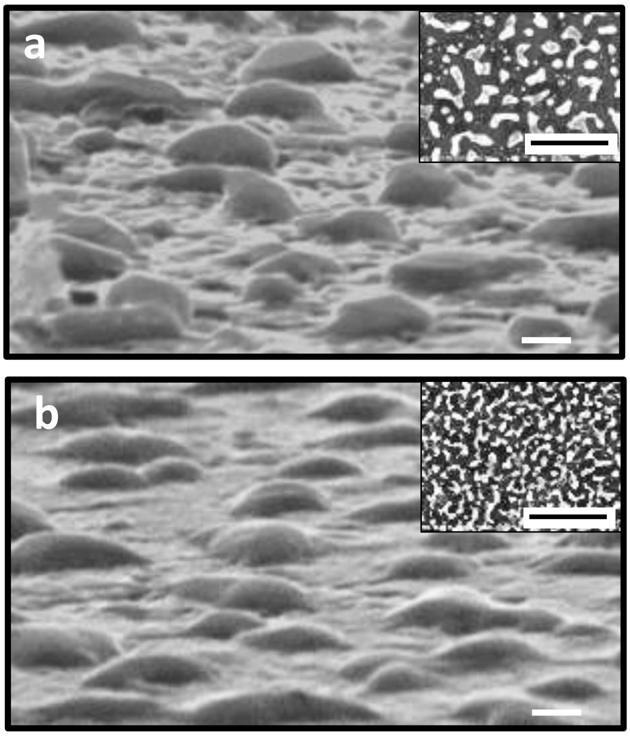

Supplementary Figure 11| Comparison of Ni nanoparticle formed by thermal and plasma CVD. SEM images of Ni nanoparticles formed by (**a**) thermal CVD and (**b**) plasma CVD. Inset shows low-magnification images. The scale bars in **a,b** and their inset show 400 nm and 5 μm, respectively.

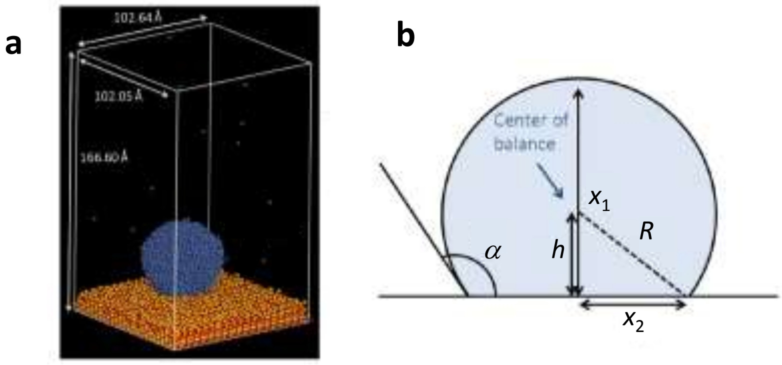

Supplementary Figure 12| Methodology of molecular dynamics simulation. (**a**) Initial configuration of molecular dynamics simulation. Blue, yellow, and red atoms represent Ni, Si, and O, respectively. (**b**) Definition of the contact angle of a droplet on the substrate assuming its shape was a sphere.

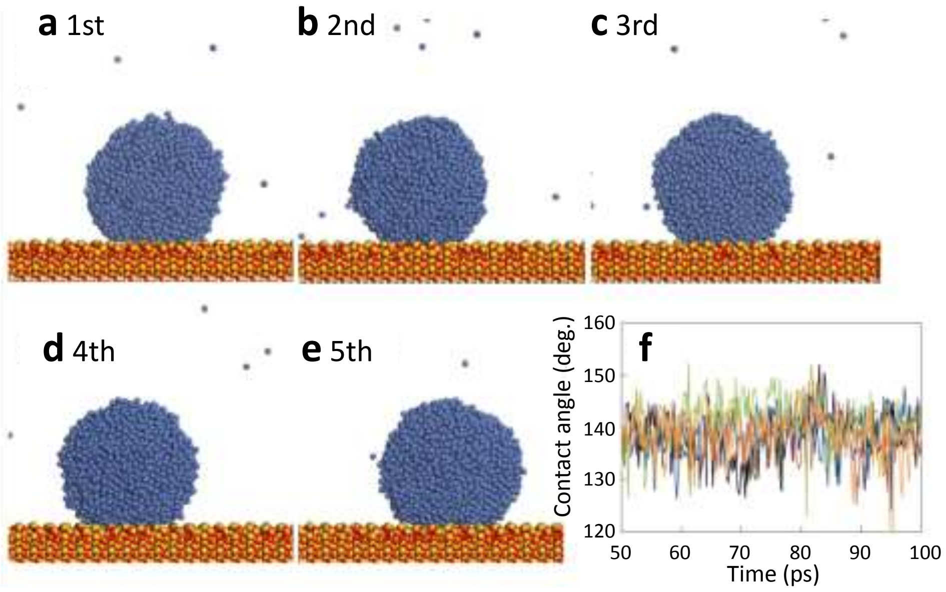

Supplementary Figure 13| Estimation of contact angle of Ni by molecular dynamics simulation. (**a-e**) Snapshots of atomic configuration of Ni nanoparticle after 100 ps calculation from five replicate calculations. (**f**) Time evolution of estimated values of contact angle of Ni nanoparticle on SiO_2 surface. Different colors represent different replicate calculations.

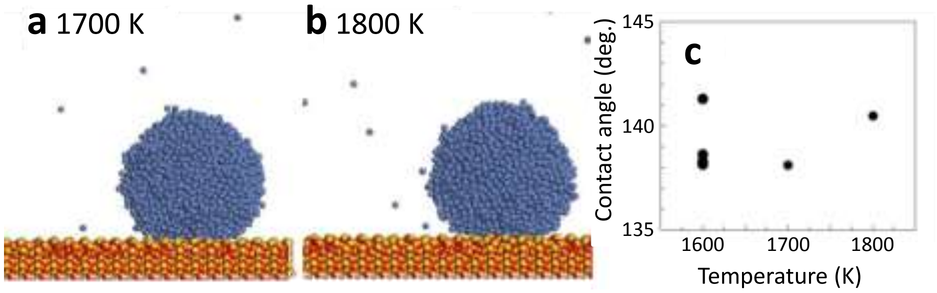

Supplementary Figure 14| Temperature dependence of contact angle. Snapshots of atomic configuration of Ni nanoparticle after 100 ps calculation for (**a**) 1700 and (**b**) 1800 K. (**c**) Estimated values of contact angle of Ni nanoparticles on SiO_2 substrate at various temperatures.

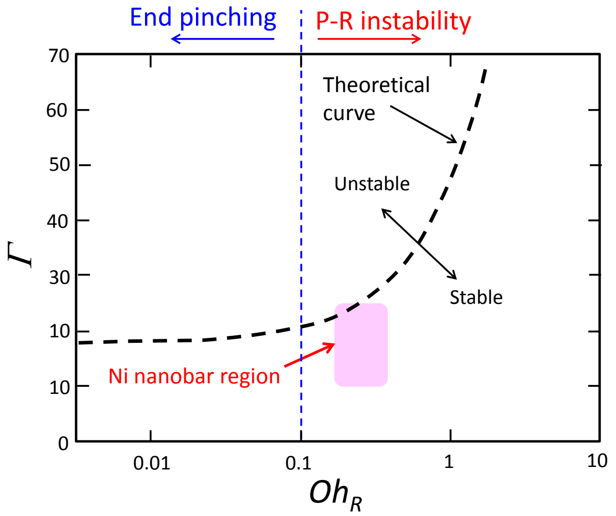

Supplementary Figure 15| Γ vs Oh_R plot. The theoretical Γ-Oh_R curve was traced from the datashown in ref[16]. The square region denotes the calculated region of Ni nanobar used in this experiment. The blue dotted line at Oh_R = 0.1 corresponds to the border between end pinching and P-R instability. Theoretical curve shows the border between stable and unstable state.

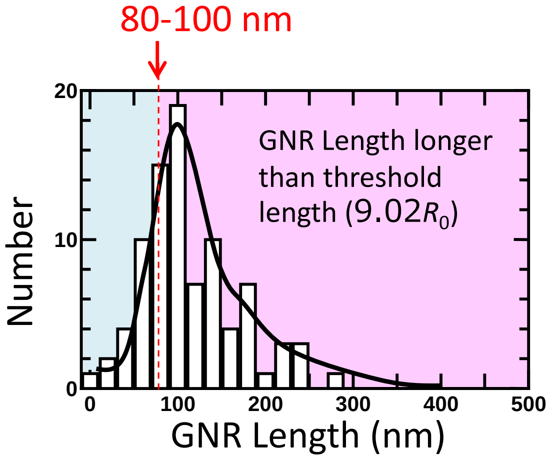

Supplementary Figure 16| GNR length histogram. Histogram of GNR length grown from 20 nm width of Ni nanobar. The dotted red line is the highlight of position of GNR Length = 80-100 nm, where the GNR length reaches to the threshold length (9.02 R_0).

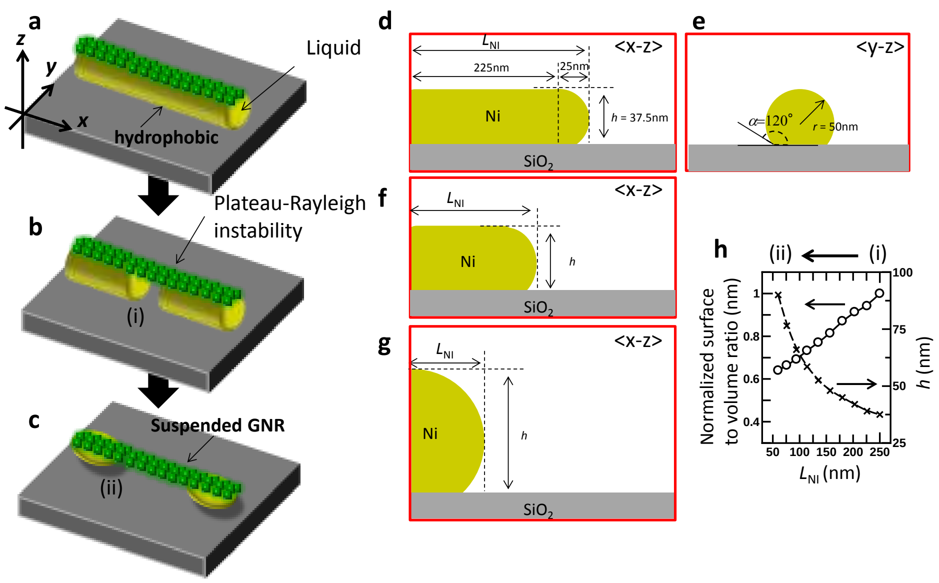

Supplementary Figure17| Formation model of suspended GNRs. (**a-c**) Schematic illustration of Ni nanobar liquid after the segregation of GNRs. (**d-e**) (**d,f,g**) *x-z* plane image and (**e**) *y-z* plane image of Ni nanobar. (**d,f,g**) The morphology change of Ni nanobar with different Ni nanobar length (L_{NI}) (**d**: long, **f**: middle, **g**: short, volume of Ni nanobar is constant). (**h**) Plot of normalized surface to volume ratio and height of Ni nanobar (h) as a function of L_{NI}. The (i) and (ii) in **h** correspond to that shown in **b** and **c**, respectively.

Supplementary Note 1. Adjustment of growth conditions for GNR synthesis

The growth conditions of GNRs was systematically adjusted by changing various parameters. Supplementary Figure 1 shows the plasma irradiation time (t_p) dependence, which is the most sensitive growth condition. The growth yield of GNRs can be high over 90% only around the specific t_p region, indicating the precise tuning of growth condition is important for the high yield synthesis of suspended GNRs with this method.

Supplementary Note 2. Analysis of suspended structure of GNRs

The suspended structures of GNRs was carefully analyzed with high resolution SEM. Supplementary Figure 2a and b,c show the low and middle magnification SEM image of GNRs array. In the high magnification image of GNR near the contact region with Ni electrode (Supplementary Figure 2d), the suspended structure of GNRs can be clearly observed, indicating suspended GNRs can be grown with this method.

Supplementary Note 3. Sub 10 nm narrow GNRs

The narrow GNR synthesis can be realized with suitable growth conditions. Supplementary Figure 3 shows typical SEM images of suspended GNR with sub 10 nm width. All of them show relatively straight and uniform color contrast image, may indicate the clean and high quality GNRs.

Supplementary Note 4. Polarized Raman measurement of GNRs

The polarized Raman features can be also observed for G-band peak. Supplementary Figure 4a, b, and c shows the intensity mapping, raw Raman scattering spectra, and polar plot of G-band intensity with parallel polarization ($I_{G//}$), respectively. Periodic oscillation of I_G can also be observed for polarization angle θ, which can be fitted by $\cos^4\theta$ function (Supplementary Figure 4d). This periodic oscillation is consistent with the previous report [1, 2].

It is known that the $I_{D//}$ over $I_{D\perp}$ can be used as an indicator for the edge roughness of graphene. The higher $I_{D//}$ over $I_{D\perp}$ratio corresponds smoother edge. This correlation can be mainly caused by the component of $I_{D\perp}$. The existence of disordered segments and microscopic misaligned segments can significantly increase $I_{D\perp}$ and hence lower the $I_{D//}$ over $I_{D\perp}$[1, 3-5]. The value of $I_{D//}$ over $I_{D\perp}$in our GNRs is relatively lower than that of the previously reported value [1], which can be explained as follows.

If the disordered segments on the edge of GNRs is the critical reason for the lower $I_{D//}$ over $I_{D\perp}$, the I_D should be independent of the polarized angle θ, which is inconsistent with our result, where periodic oscillation of I_D against to the θ can be appeared (Fig. 3c). Also, the ratio of $I_{D//}$ over $I_{G//}$ should become large for such disordered edges. This is also inconsistent with our result (low $I_{D//}$ over $I_{G//}$). These show that the edge of GNRs grown by our method should be at least smoother than that of lithographically etched GNRs, which is known as the rough edge structures. Then, the possible two reasons can be given for the lower $I_{D//}$ over $I_{D\perp}$ in our sample even with the preferential near zigzag edge structures.

The first one is microscopic misalignment of edges. GNRs grown in our study has well aligned structures between Ni electrode as can be seen in Fig. 2d and e. However, it is very difficult to identify the microscopic alignment with atomic resolution of GNR edge along the whole GNR edge. In our GNRs, the width of GNR near the contact region of Ni can be slightly larger than the middle region, indicating the width of GNR changed along the same GNR (Supplementary Figure 5a). This show the alignment angle of zigzag edge may be partially different, which can cause the increase of $I_{D\perp}$, resulting in the low $I_{D//}$ over $I_{D\perp}$(Supplementary Figure 5b). Since the $I_{D//}$ in zigzag edge is weak or null, other orientation such as armchair may be formed at such partial misaligned regions.

The second one is amorphous carbon residue. Since we used as grown GNRs for all of the measurements, certain amount of amorphous carbon may be existed on the SiO_2 substrate, Ni surface, and on GNRs, which can be detected as non-polarized component of D-band and it can lower the $I_{D//}$ over $I_{D\perp}$.

Based on these reasons, we think that the near zigzag orientation should be dominant, which can be identified from lower $I_{D//}$ over $I_{G//}$. However, microscopic alignment angle of the near zigzag edge can be varied depending on the position of same GNR. Certain amount of amorphous carbon residue can be deposited on the

sample during the CVD process. These can be the reason for the relatively low $I_{D//}$ over $I_{D\perp}$ in our GNR sample.

Supplementary Note 5. Suspended GNR transistor with side gate operation

The side gate operation of suspended GNRs can be observed in other devices. Supplementary Figure 6a and b shows a typical scanning electron microscopy (SEM) image of a suspended GNR field effect transistor (FET) with side gate structures. The width of the GNR is about 37 nm. The charge neutral point in the source–drain current (I_{ds}) vs. back gate bias voltage (V_{bg}) curve can be modulated by the side gate bias voltage (V_{sg}) (Supplementary Figure 6c), indicating that a locally addressable suspended FET can be fabricated, just as in the results shown in the main text (Fig. 4).

Supplementary Note 6. GNR synthesis from Ni thin film

The suspended GNR and graphene synthesis could also be realized using a thin (approximately 80 nm) Ni film. The suspended structures of the GNR (Supplementary Figure 7a and b) and graphene (Supplementary Figure 7c and d) were grown using the method discussed in the main text. The typical Raman scattering spectra (Supplementary Figure 7e) also showed the synthesis of monolayer graphene with the suspended structures.

Supplementary Note 7. Analysis of Ni film deformation

An estimation of the depression rate of the surface coverage of Ni (ΔS_{Ni}) was carried out using a combination of SEM measurement and automated data analysis with image processing. The SEM image of the Ni thin film after CVD showed deformed particle-like shapes, as shown in Supplementary Figure 8a. The SEM image could be converted into a black and white color image (Supplementary Figure 8b), which made it possible to estimate the quantitative areas of the white (Ni) and black (SiO_2) regions. An accurate ΔS_{Ni} value could be estimated using the areas of the white regions before and after the CVD.

Supplementary Note 8. Phase diagram calculation

The phase diagram of the Ni-C binary system consists of a simple eutectic reaction with two terminal phases, fcc-Ni and graphite [6]. A thermodynamic assessment of this system was carried out by Guillermet based on the CALPHAD method [7]. In this study, the phase diagram was calculated using the model of Ref. [7], which is explained below.

The phase equilibria of this system can be described by the Gibbs energies for the liquid, fcc, and graphite phases. The regular solution model was applied to the liquid phase. The molar Gibbs energy of the liquid phase $G_{\mathrm{m}}^{\mathrm{liquid}}$ is expressed as follows:

$$G_{\mathrm{m}}^{\mathrm{liquid}} = x_{\mathrm{Ni}} G_{\mathrm{Ni}}^{0,\mathrm{liquid}} + x_{\mathrm{C}} G_{\mathrm{C}}^{0,\mathrm{liquid}} + RT\left(x_{\mathrm{Ni}} \ln x_{\mathrm{Ni}} + x_{\mathrm{C}} \ln x_{\mathrm{C}}\right)$$

$$+ x_{\mathrm{C}} x_{\mathrm{Ni}} \sum_{v} L_{\mathrm{C,Ni}i}^{\mathrm{liquid}v} \left(x_{\mathrm{C}} - x_{\mathrm{Ni}}\right)^{v}, \qquad \text{(Supplementary Equation 1)}$$

where x_i with i = Ni and C are the mole fractions of Ni and C, respectively; R is the gas constant; and T is the temperature. $G_i^{0,\mathrm{liquid}}$ is the Gibbs energy function for the pure element i in the liquid phase, which was taken from the SGTE database [8], and $L_{\mathrm{C,Ni}}^{\mathrm{liquid}v}$ is the vth Redlich-Kister parameter.

The fcc phase was modeled using a two sublattice model, the formula of which is given as Ni(C,Va), where Va represents the vacancy. The Gibbs energy of this phase per mole of formula $G_{\mathrm{m}}^{\mathrm{fcc}}$ is then given by

$$G_{\mathrm{m}}^{\mathrm{fcc}} = y_{\mathrm{C}} G_{\mathrm{Ni:C}}^{\mathrm{fcc}} + y_{\mathrm{Va}} G_{\mathrm{Ni:Va}}^{\mathrm{fcc}} + RT\left(y_{\mathrm{C}} \ln y_{\mathrm{C}} + y_{\mathrm{Va}} \ln y_{\mathrm{Va}}\right)$$

$$+ y_{\mathrm{C}} y_{\mathrm{Ni}} \sum_{v} L_{\mathrm{Ni:C,Va}}^{\mathrm{fcc},v} \left(y_{\mathrm{Ni}} - y_{\mathrm{Va}}\right)^{v}, \qquad \text{(Supplementary Equation 2)}$$

where y_{C} and y_{Va} are the site fractions of C and the vacancy, respectively, on the second sublattice. $G_{\mathrm{Ni:Va}}^{\mathrm{fcc}}$ is the Gibbs energy function for Ni in the fcc phase, and it was taken from the SGTE database [8]. $G_{\mathrm{Ni:C}}^{\mathrm{fcc}}$ is the Gibbs energy for a fictitious compound, and

$L^{\mathrm{fcc},v}_{\mathrm{Ni:C,Va}}$ is the vth interaction parameter between C and the vacancy on the second sublattice. No solubility was taken into account for the graphite phase, and the SGTE database [8] was employed to describe the thermodynamic state of the graphite. All of the other parameters described above were reported in Ref. [7].

The phase diagram was calculated on the basis of the common tangent rule using the above-mentioned Gibbs energies. The calculated phase-diagrams are shown in Supplementary Figure 9. The dashed lines in Supplementary Figure 9a and b indicate the phase boundaries between the liquid and fcc, which were calculated by suspending the precipitation of the graphite.

Note that these Gibbs energy models, and thus the calculated phase diagram, can be applied only to the discussion for the bulk states. As described in the main text, the nanoscale effect, i.e., capillary effect, should not be negligible for the description of the thermodynamic state of the nanobar. Although the CALPHAD method has been extended to deal with the phase equilibria for a nanoparticle by including the surface energy contribution [9], the present discussion is based on thermodynamic states for the bulk states because of a lack of the information required to calculate the surface energy contribution. According to the Monte Carlo study on the phase equilibria of Ni-C nanoclusters, the qualitative trend of phase equilibria in Ni-rich part is quite similar to those of the bulk states [10]. Hence, the phase diagram shown in Supplementary Figure 9 carries qualitative information, which is sufficient for the present purpose.

Supplementary Note 9. Ni nanobar after annealing without carbon supply

Simple annealing under various temperature conditions (550~900 °C) was carried out using Ni nanobar structures (Supplementary Figure 10). The annealing was performed under the Ar atmosphere with 250 Pa, which is same with that of CVD. The Ni nanobar structures could not be observed after the annealing over 600 °C, indicating that the Ni nanobar should have been partially melted and dewetted. Although this temperature (600 °C) is lower than the melting point of bulk Ni crystal (approximately 1455 °C), this result can be explained by the nanoscale effect, where the melting point can decrease by several hundred degrees compared with the bulk crystal.

Supplementary Note 10. Comparison of Ni nanoparticles fabricated by thermal CVD and plasma CVD

The wettability of Ni nanoparticles after thermal (Supplementary Figure 11a) and plasma CVDs (Supplementary Figure 11b) was analyzed using conventional and tilted SEM measurements. The contact angles of Ni nanoparticles after the thermal and plasma CVDs clearly differed, as discussed in the main text (Fig. 6b and c).

Supplementary Note 11. Molecular dynamics simulation of Ni nanoparticle on SiO_2 substrate

Classical molecular dynamics simulations were performed to investigate the wettability of a Ni nanoparticle on a SiO_2 substrate using MS ForcitePlus in Materials Studio 8.0 [11]. The Condensed Phase Optimized Molecular Potentials for Atomic Simulation Studies II (COMPASS II) was employed for the interatomic potential [12, 13]. The cutoff distance for short-range interactions was set to 12.5 Å. The Coulomb interactions were calculated using the Ewald method. The velocity Verlet algorithm was used to integrate the classical equation of motion with a time step of 2.0 fs. A Nose thermostat was applied to control the temperature. Supplementary Figure 12a shows the initial configuration of the calculation system, which was prepared as follows. First, a liquid droplet of Ni were prepared by heating fcc Ni crystals with 4394 atoms (13 × 13 × 13 unit cells) for 300 ps at 1600 K. The obtained liquid Ni droplet was placed onto the (110) surface of SiO_2 consisting of 3420 Si and 6384 O atoms. Geometric optimization was carried out in advance of the main calculations by performing a complex calculation involving the steepest descent and conjugate gradient methods with a total of 5000 steps. From the obtained initial configuration, the main calculation was performed during 100 ps at 1600 ps. The bottom layer of SiO_2 was fixed during the main calculation. Five replicate calculations were performed to gather statistics.

The data obtained from last 50 ps were used to estimate the contact angle of the Ni nanoparticle. The contact angle was estimated on the assumption that the surface of the droplet was spherical [14, 15]. As defined in Supplementary Figure 12b, the contact angle a was determined from the radius R and height of the center of the droplet h as follows:

$$\cos a = -\frac{h}{R}. \qquad \text{(Supplementary Equation 3)}$$

Here, R and h were estimated from the height of the droplet x_1 and the radius of the cross-sectional circle at the bottom of the droplet x_2 as follows:

$$R = \frac{x_1^2 + x_2^2}{2x_1}, \qquad \text{(Supplementary Equation 4)}$$

$$h = \frac{x_1^2 - x_2^2}{2x_1}. \qquad \text{(Supplementary Equation 5)}$$

Therefore, a can be determined from x_1 and x_2 as follows:

$$a = \cos^{-1}\left(\frac{x_2^2 - x_1^2}{x_2^2 + x_1^2}\right). \qquad \text{(Supplementary Equation 6)}$$

Supplementary Figure 13a-e shows snapshots of the atomic configuration of Ni nanoparticles at 100 ps from replicate calculations. There was no significant difference in the structures of the Ni nanoparticles among the five replicate calculations. It is obvious from the snapshots that the Ni nanoparticle is hydrophobic on the SiO_2 substrate, with a contact angle larger than 90°. Supplementary Figure 13f shows the time evolution of the contact angle of the Ni particle on the SiO_2 surface from the five replicate calculations. It was found that the contact angle converged at around 140° during the latter half of all five replicate calculations. The contact angle average over time is shown in Fig. 6g. The contact angle was finally estimated to be 138. 93 ± 1.2°.

Moreover, the temperature effect on the contact angle of the Ni nanoparticle on the SiO_2 substrate was investigated by further calculations for 1600 and 1700 K. Supplementary Figure 14a and b shows snapshots of the atomic configurations of Ni nanoparticles at 100 ps from the calculations for 1700 and 1800 K. The estimated values of the contact angles of these nanoparticles are summarized in Supplementary Figure 14c. The values for 1600 K are also plotted for comparison. There is no significant difference in the snapshots and estimated values of the contact angle among the cases for 1600 and 1800 K. Therefore, it was concluded that the contact angle had no temperature dependence in the examined temperature range.

Supplementary Note 12. Plateau-Rayleight instability

Supplementary Figure 15 shows the Γ vs Oh_R plot. The original plot was reported in ref [16]. We added the square region in this Supplementary Figure 15,

denoting the Ni nanobar region used in our experiment. Since Oh_R of Ni nanobar is over 0.1 (0.215−0.34), the dominant breaking mechanism of Ni nanobar should be P-R instability.

The distribution of GNR length was measured over 40 GNRs (Supplementary Figure 16). The almost all of GNR length is longer than 80-100 nm, which is very close to the threshold length (90.2 nm) calculated with the equation for Plateau-Rayleight instability [17].

Supplementary Note 13. Growth model of suspended GNRs

The surface to volume ratio was calculated for the Ni nanobar with same geometry used in the experiment (Supplementary Figure 17). It is found that the surface to volume ratio decrease about 64% (Supplementary Figure 17h) by changing the morphology from just after the breaking of Ni nanobar (Supplementary Figure 17 b,d) to particle like shape (Supplementary Figure 17c,g). This show the morphology change of Ni nanobar after the segregation of GNRs is possible to satisfy the minimum surface energy, resulting in the formation of suspended GNR between Ni electrodes.

Supplementary References

1. Xie, L., *et al.* Graphene Nanoribbons from Unzipped Carbon Nanotubes: Atomic Structures, Raman Spectroscopy, and Electrical Properties. *J. Am. Chem. Soc.* **133**, 10394‑10397 (2011).

2. Cançado, L. G., Pimenta, M. A., Neves, B. R. A., Dantas, M. S. S. & Jorio, A. Influence of the Atomic Structure on the Raman Spectra of Graphite Edges. *Phys. Rev. Lett.* **93**, 247401 (2004).

3. Casiraghi, C., *et al.* Raman Spectroscopy of Graphene Edges. *Nano Lett.* **9**, 1433-1441 (2009).

4. Yang, R., Shi, Z., Zhang, L., Shi, D. & Zhang, G. Observation of Raman G-Peak Split for Graphene Nanoribbons with Hydrogen-Terminated Zigzag Edges. *Nano Lett.* **11**, 4083-4088 (2011).

5. Gong, Y. P., *et al.* Electronic transport properties of graphene nanoribbon arrays fabricated by unzipping aligned nanotubes. *Phys. Rev. B* **87**, 165404 (2013).

6. Singleton, M. F., Nash, P., Binary Alloy Phase Diagrams (Ed. By B. Massalski, H. Okamoto, P.R. Subramanian, L. Kacprzak), vol. 1, ASM International, Materials Park, OH, 866-867 (1992).

7. Guillermet, A. F. Thermodynamic Properties of the Fe-Co-Ni-C System. *Z. Metallkd.* **79**, 524-536 (1988).

8. Dinsdale, A. T. SGTE data for pure elements. *Calphad* **15**, 317-425 (1991).

9. Park, J. & Lee, J. Phase diagram reassessment of Ag-Au system including size effect. *Calphad* **32**, 135-141 (2008).

10. Brillo, J. & Egry, I. Surface tension of nickel, copper, iron and their binary alloys. *J. Mater. Sci.* **40**, 2213-2216 (2005).

11. BIOVIA Materials Studio 8.0, Dassault Systemes Biovia, 2015.

12. Sun, H. An ab initio force-field optimized for condensed-phase applications overview with details on alkane and benzene compounds. *J. Phys. Chem. B* **102**, 7338-7364 (1998).

13. Sun, H. & Rigby, D. Polysiloxanes: ab initio force field and structural, conformational and thermophysical properties. *Spectrochim. Acta Part A* **53**, 1301-1323 (1997).

14. Shibuta, Y. & Suzuki, T. Phase transition in substrate-supported molybdenum nanoparticles: a molecular dynamics study. *Phys. Chem. Chem. Phys.* **12**, 731-739 (2010).

15. Shibuta, Y. & Suzuki, T. Effect of wettability on phase transition in substrate-supported bcc-metal nanoparticles: a molecular dynamics study. *Chem. Phys. Lett.* **498**, 323-327 (2010).

16. Driessen, T. W., Jeurissen, R. J. M., Wijshoff, H., Toschi, F. & Lohse, D. Stability of viscous long liquid filaments. *Phys. Fluids* **25**, 062109 (2013).

17. Bush, J. W. M. *MIT Lecture Notes on Surface Tension*. lecture 5 (Massachusetts Institute of Technology, 2004). http://www.google.co.jp/url?sa=t&rct=j&q=&esrc=s&source=web&cd=1&cad=rja&uact=8&ved=0ahUKEwjo0-mr45fMAhVEraYKHdC3BioQFggbMAA&url=http%3A%2F%2Fweb.mit.edu%2F2.21%2Fwww%2FLec-notes%2FSurfacetension%2FLecture5.pdf&usg=AFQjCNGGd0OR0FgjyEJOHsyB1NJiTNksDg&sig2=Bmttp-cTFEWNx7nQECICvw.
